# Genetic determinants and absence of breast cancer in Xavante Indians in Sangradouro Reserve, Brazil

**DOI:** 10.1038/s41598-023-28461-y

**Published:** 2023-01-26

**Authors:** Yan Zhou, Jose Russo, José Rueff, Marcelo A. M. Pires, Guilherme Bezerra de Castro

**Affiliations:** 1 Biostatistics and Bioinformatics Facility, Fox Chase Cancer Center, Philadelphia, PA USA; 2 The Irma H Russo, MD-Breast Cancer Research Laboratory, Fox Chase Cancer Center, Philadelphia, PA USA; 3 Center for Toxicogenomics and Human Health, Nova Medical School, Universidade Nova de Lisboa, Lisbon, Portugal; 4 Molecular Cancer Research Center, Cuiabá, Mato Grosso Brazil; 5 Molecular Cancer Research Center, P.O.Box 1559, Woking, GU22 2WN UK

**Keywords:** Cancer, Genetics, Oncology, Risk factors

## Abstract

Genetic compositions of distinct human populations are different. How genomic variants influence many common and rare genetic diseases is always of great medical and anthropological interest, and understanding of genetic architectures of population groups in relation to diseases can advance our knowledge of medicine. Here, we have studied the genomic architecture of a group of Xavante Indians, an indigenous population in Brazil, and compared them with normal populations from the 1000 Genomes Projects. Principal component analysis (PCA) indicates that the Xavante Indians are genetically distinctive when compared to other ethnic groups. No incidence of breast cancer cases has ever been reported in the population, and polygenic risk analysis indicates extremely low breast cancer risk in this population when compared with germline TCGA (The Cancer Genome Atlas) breast cancer normal control samples. Low germinal mutation burden among this population is also observed. Our findings will help to deepen the understanding of breast cancer and might also provide new approaches to study the disease.

## Introduction

According to the World Health Organization, in 2020^1^ there were 2.3 million women diagnosed with breast cancer and 685,000 deaths globally, as well as 7.5 million living with the disease over the past five years. This is, therefore, a global health issue, with incidences worldwide varying in accordance with life expectancy, cultural behavior and level of development of the corresponding countries or regions.

Indeed, an observation by Guilherme Bezerra de Castro^2^ brought attention to an unusual observation^3^, that there are no reported incidences of breast cancer among Xavante Brazilian Indians. But other types of cancers do exist in this population such as cervical, lung, stomach and leukemia. The Xavante are Brazilian Indians, a Macro-Jê linguistic trunk of the Tupy branch, who were linked to the Asiatic population that migrated to the Americas through the Bering Strait more than 15,000 years ago by tracking Y chromosome studies^4^. These primitive people, after descending the Pacific coast along the Andes, later populated the Caribbean Islands and reentered the continent by the coast of Guyana. From here, this group gradually diverged from the other people of the Americas, such as the Mayas and Aztecs from Central America, and Incas from South America. The Brazilian Indians then penetrated the Amazon rainforest, which 12,000 years ago was a large savanna in its central area^5^. At that point they divided into two branches: the Tupy living across the Atlantic coast, and the Macro-Jê branch settling in the Central Region of Brazil who maintained the lifestyle of nomadic hunter-gatherers until today. The latter maintained a well-preserved culture^6^. Currently, they are the largest indigenous group^7^ with almost 22 thousand Xavante, 50% being female, living in 11 reserves in Mato Grosso State, with a population growth of more than 5% per year^8^. In 1993, Dr. Castro made an observation that this population group does not appear to develop breast cancer during their lifetime after working with the Xavante for 10 years. Since then, there have been no occurrences contradicting our observation^9^, and no study about breast cancer in Brazilian indigenous women has been published in the country to the present day.

Although analysis of the genetic makeup of the different populations in relation to cancer incidence is yet to be fully discerned, we carried out the current study to investigate the genetic characteristics of the Xavante to attempt to understand the observation on the lack of breast cancer incidence in this population and to provide comparisons with other ethnic groups from the 1000 Genomes Project, which include European, African, Asian and Hispanic populations.

## Methods

### Ethics statement

Authorization from Fundação Nacional do Índio (FUNAI) was acquired after approval from the Research Ethics Committee of the Faculty of Medicine in the Federal University of Mato Grosso (UFMT), and the National Commission of Research Ethics (authorization #1004/2001). Written consents, which were recorded and archived, were acquired from all subjects prior to the study either by the individual or their legal guardian in the case of minors, as well as the use of any documents necessary for the study of the Xavante population.

### Sample selection

Out of approximately 500 women in the Sangradouro Reserve (located 270 km east of Cuiabá, the capital of Mato Grosso state, in central Brazil), 182 volunteered for the project with a median age of 27 and life expectancy of 61.7 years^10^. We then distributed a questionnaire about comorbidity, number of offspring, breastfeeding, height, weight, and breast pathology. Blood samples from 14 of these women were used for exome-sequencing for study of this homogenous ethnic population. This cohort was selected because they are the least mixed ethnic group among those living close to cities, providing good access for the researchers to support the study, and their demographic information is listed in Supplementary Table 1. The genetic variants of this cohort were then assembled and analyzed together with the 1000 Genomes Project Phase 3 genomic variation data which includes 2542 control samples (522 white, 671 black, 515 East Asian, 348 Hispanic and 492 South Asian).

### Sample collection

All DNA samples were extracted from 250 μl of whole blood using a commercially available kit according to the manufacturer's instructions (QIAamp DNA extraction kit; Qiagen, Hilden, Germany). After extraction, the determination of the concentration of all DNA samples was carried out using the PicoGreen dsDNA quantification reagent (Molecular Probes, Eugene, OR, USA) in an Anthos Zenyth 3100 (Anthos-Labtec Instruments GmbH, Austria). The linearity of the method was verified for the high-range standard curve (2 μg/ml of Lambda DNA standard) according to the manufacturer's recommendations prior to determination the DNA concentration.

### Whole-exome sequencing

Genomic DNA from 14 female Xavante from the Mato Grosso State was prepared. A total amount of 1.0 μg genomic DNA per sample was used as input material for the DNA library preparation. Sequencing libraries were generated using Agilent SureSelect Human All Exon kit (Agilent Technologies, CA, USA) and were sequenced on an Illumina NextSeq 500 system (Illumina, San Diego, CA) by Novogene Co. Ltd (Chula Vista, California, USA). Reads were mapped to the human genome reference (GRCh38) with BWA^11^, HaplotypeCaller from GATK best practices workflow^12^ was used for variant calling, and ANNOVAR^13^ was used for variant annotation and effect prediction. Only variants classified as “PASS” were considered for analyses. Variants that were not reported in dbSNP v147 and did not have population frequencies reported in 1000 Genome phase 3 data were considered novel. The functional consequences of nonsynonymous SNVs and splice variants were predicted using PolyPhen-2 (Polymorphism Phenotyping v2), SIFT, LRT, Mutation Taster, Mutation Assessor, and CADD^14^.

### Analysis of population admixture

Genomic variation data from the 1000 Genomes Project Phase 3 collection^15^, which includes 2548 samples from 26 populations were used for population admixture analysis together with our 14 samples. A total of 291,984 SNPs shared among these populations and also present in at least 7 of our samples were used to generate principal component analysis (PCA) plots after applying a linkage disequilibrium based variant pruner as implemented in PLINK^16^. Supplementary Fig. 2 shows the estimated individual ancestry proportions for K = 4 to K = 11 (parameter K describes the hypothesized number of subpopulations that make up the total population) in all 1000 Genome samples and the 14 Xavante samples using ADMIXTURE^17^. The fit of different values of K was assessed using cross-validation (CV) procedures, and K = 9 showed the lowest CV error (Supplementary Fig. 3).

### Polygenic risk estimation

Khera et al. have developed a genome-wide polygenic score for breast cancer risk estimation which comprises 5218 common (allele frequency > 1%) variants^18^. This score is based on association statistics for millions of variants derived from previously published genome-wide association studies of up to 105,974 individuals with breast cancer and 122,977 control subjects^19^. We applied this computational algorithm to generate a polygenic score for each sample that integrates the cumulative impact of all available variants. We also analyzed germline mutation data from normal blood samples from 10 lobular and 10 ductal normal blood samples from the TCGA (The Cancer Genome Atlas) breast cancer dataset as comparison. To minimize potential confounding from whole-genome sequencing performed in separate batches for the Xavante and TCGA cohorts, we assembled a single joint variant call set across all samples in this analysis starting from raw reads. After application of stringent sequencing quality control parameters, 2171 out of the 5218 (41.6%) variants were available for scoring, and this polygenic score was calculated in each of our samples as well as the TCGA breast cancer normal control samples.

### Mutation load analysis

Genomic germline variation data which include SNVs and short indels from our cohort, 1296 normal female samples from the 1000 Genomes Project, and 200 randomly selected TCGA normal blood samples (100 ductal and 100 lobular cancer subtype) were analyzed and annotated together. Exonic or splicing variants which have less than 1% population frequency and which were predicted to be damaging by at least 2 methods listed above were retained. We then compared the number of genes which carry these potenitally damaging variants among all sample populations. Since we were also interested in looking at relatedness to breast cancer risk, we further examined the mutation status of known breast cancer risk genes in all samples using the list recommended by National Breast Cancer Foundation^20^, which includes *ATM, BARD1, BRCA1, BRCA2, BRIP1, CASP8, CDH1, CHEK2, CTLA4, CYP19A1, FGFR2, H19, LSP1, MAP3K1, MRE11A, NBN, PLAB2, PTEN, RAD51, STK11, TERT* and *TP53* genes.

### Statistical analysis

2-sided Wilcoxon–Mann–Whitney tests (5% type I error) were used to compare of polygenic risk score and mutation load analysis between groups. Analyses were performed using R.

## Results

### Demography of our Xavante samples

The data we have collected showed that these women, on average, begin menarche at 12 years old, have their first child at age 15, breastfeed for two years, have seven children during the course of their reproductive lives (data not shown) and have low mammary pathology which is consistent with published demographic and behavioral data^21^ about this population. Blood tests were also performed for biochemistry, hormonal and complete blood count which revealed an abnormal low hormonal level and high level of glycemia (data not shown) which is also observed by a recent publication^22^.

### Sequence variation of Xavante samples

The mean coverage for all sequenced genomes was ~ 100× (Supplementary Fig. 1). On average, 99.3% of the bases in the target region of human exome (build 38) were covered by at least 5 reads (Supplementary Table 2). The mean number of single-nucleotide variants (SNVs) was 368,309 for the 14 samples. Overall, 5.4% of SNVs were located in coding regions, 2.2% were missense, and 0.015% were nonsense (Supplementary Table 3). Interestingly, the proportion of homozygous SNVs was around 75% (Supplementary Table 4). The distribution of insertions and deletions is described in Supplementary Table 5.

### Population admixture

**Figure 1:**
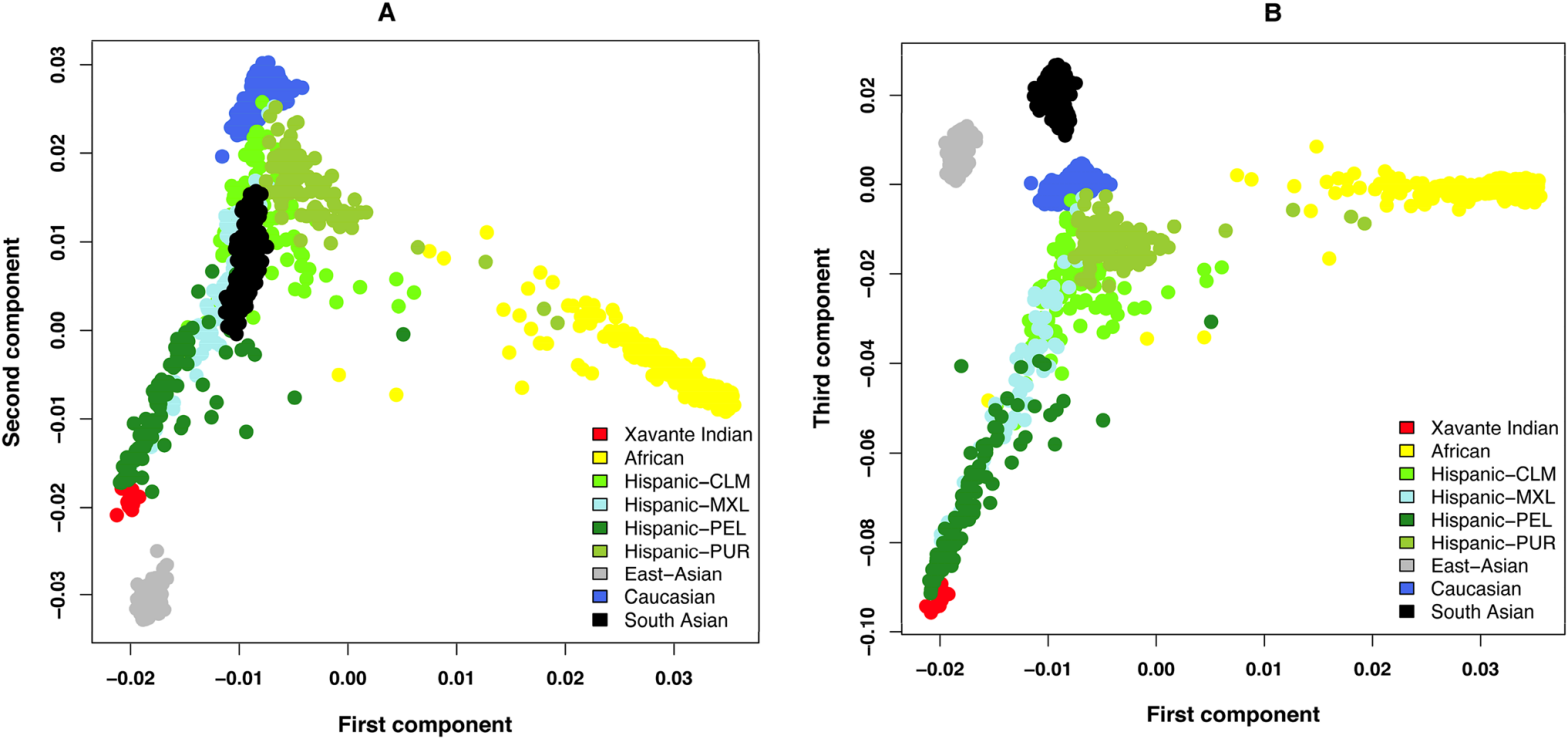
Princiapl Component Analysis (PCA) plots of the admixture analysis of the 14 Xavante Indian samples together with the 2548 normal samples from 1000 genome project. 291,984 SNPs shared among all populations were used. (**A**) PCA plot for components 1 and 2. (**B**) PCA plot for components 1 and 3. This confirms that the populations were well matched with respect to genetic background and Xavante Indian is a unique genetic group.

**Figure 2:**
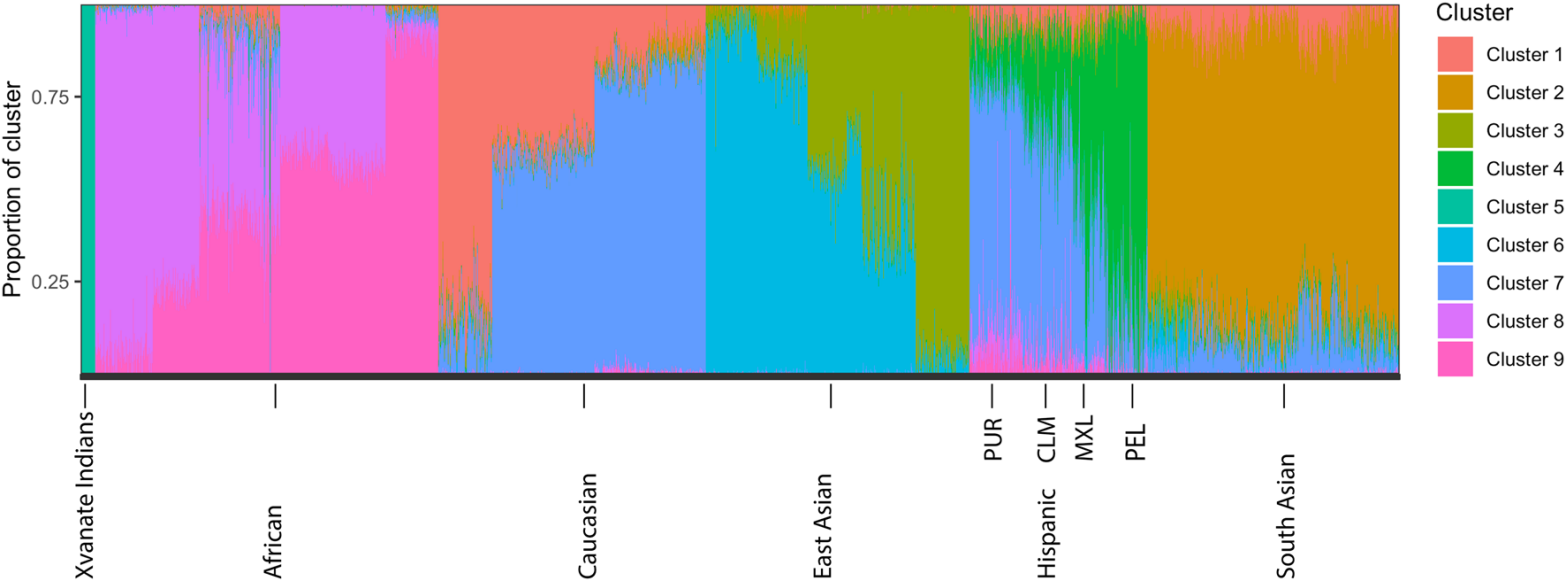
Global ancestry proportion of Xavante Indian samples and reference populations from 1000 genome project assuming K = 9 when analyzed using ADMIXTURE. Each color represents a unique genetic cluster identified by ADMIXTURE. *PUR* Puerto Ricans from Puerto Rico, *CLM* Colombians from Medellin, Colombia, *MXL* Mexican Ancestry from Los Angeles USA, *PEL* Peruvians from Lima, Peru.

Genome variation data from our cohort and 1000 Genomes reference populations were used to generate PCA plots. Figure 1A shows that components 1 and 2 distinguish Africans, Europeans and East Asians from Mexicans, with 14 Xavante clustered close to the latter group. Moreover, component 3 (Fig. 1B) further separates South Asians, showing our cohort as a distinct population clustered next to the Hispanic populations, among which the PEL (Peruvians from Lima, Peru) subpopulation is the closest to the Xavante. This ancestry proportion analysis using ADMIXTURE revealed that our 14 samples have a very distinct genetic profile when compared to all other control groups (Fig. 2). Xavante samples have fairly homogeneous ancestry composition and also formed a very unique genetic cluster (green bar on the far left) which was distinctive when compared to all other populations.

### Polygenic risk score

**Figure 3:**
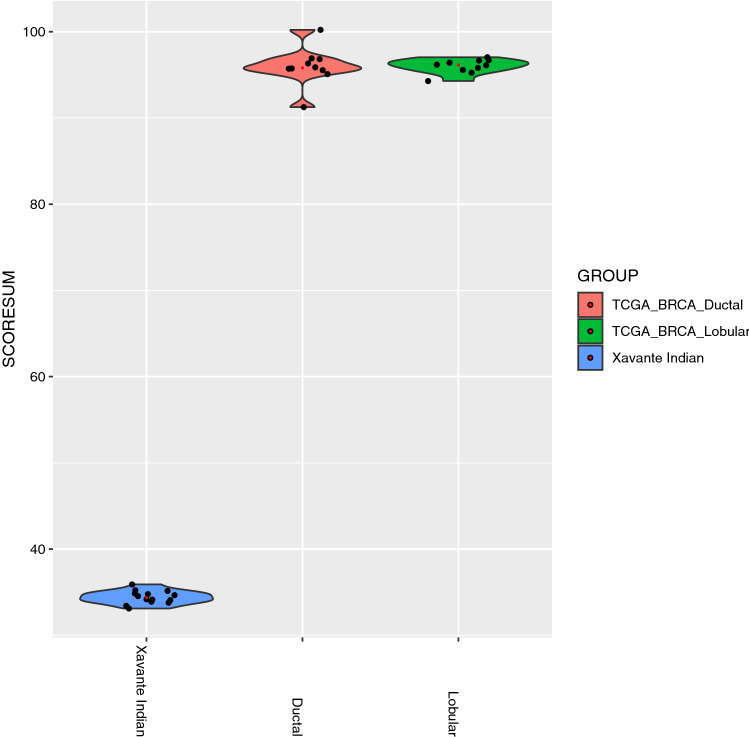
Violin plot of calculated polygenic risk scores of Xavante Indian samples and TCGA normal breast cancer control samples to compare the genetic risk for breast cancer of Xavante Indians and TCGA breast cancer normal controls. Each dot represents the polygenic risk score of one sample. Wilcoxon–Mann–Whitney test p value < 0.0001 indicated extremely significant difference between groups.

We next calculated a polygenic risk score comprising 2171 variants in all 14 Xavante samples as well as 20 TCGA breast cancer normal blood samples using the algorithm developed by Khera et al.^18^. As expected, samples from our cohort of healthy Xavante women displayed low polygenic risk when compared to TCGA breast cancer normal blood samples (Fig. 3) (P < 0.0001). These results provide preliminary evidence that the absence of observed breast cancer in this indigenous group may, in part, result from their genomic characteristics.

### Mutation load across Exome

**Figure 4:**
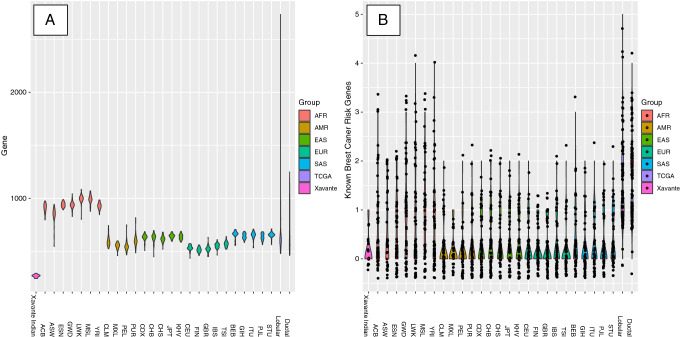
Violin plot of number of mutated genes affected by rare damaging variants in different populations which include Xavante Indians, normal populations from 1000 Genome Project and normal samples from TCGA breast cancer dataset. (**A**) Number of mutated genes in the whole exome (p < 0.0001 for all groups against Xavante Indians); (**B**) Number of mutated genes in the known breast cancer risk genes (p < 0.0001 for Xavante Indians vs. TCGA breast normal control groups).

We were also interested in finding out whether Xavante women have a lower germline mutation rate in general compared to other populations. We analyzed the rare mutations with potentially damaging effect, then compared the number of genes whose function would likely be affected by these mutations. They seem to have significantly lower number of mutated genes (mean = 268.93, standard deviation = 13.74) when compared to the 1000 Genomes reference populations and TCGA breast cancer samples (Fig. 4A) (P < 0.0001 for all pairwise comparisons against our cohort as shown in Supplementary Table 6). We then explored the mutation status of genes known to be linked to breast cancer risk. The Xavante cohort still has the lowest mutation burden on the breast cancer risk genes, while TCGA breast cancer normal blood samples have the most mutated breast cancer risk genes (Fig. 4B, Supplementary Table 7). In this comparison, only TCGA lobular and ductal normal control samples are significantly different from the Xavante with P < 0.0001.

### Identification of variant alleles with biomedical relevance

We analyzed all exomes of our cohort for the presence of potentially pathogenic variants as defined by the publication of the American College of Medical Genetics and Genomics (ACMG)^23^. We looked for incidental findings among the 56 genes recommended by the ACMG and found a total of 8 non-synonymous variants in the exomes of our samples which have less than 1% minor allele frequencies in the 1000 Genomes Project (Phase3). Six of these variants were detected only in one of our samples, and 1 variant in the *DSP* gene (rs368802003) was detected in 2 samples. These 7 variants either lacked ClinVar annotation or were annotated as having benign or unknown effect by ClinVar. A *MYBPC3* variant (rs11570112) was detected in 5 of our samples with possible pathogenic effect (Supplementary Table 8). This gene is reported by ACMG to be related to hypertrophic cardiomyopathy and dilated cardiomyopathy^24^. In the last few decades important changes have occurred in the lifestyle and epidemiological profile of this population. Recent studies have indicated high cardiovascular risk according to several indicators evaluated^25^, and a high incidence of diabetes^26^. However, high incidence of hypertrophic cardiomyopathy has not been observed in this population^23^. Since the phenotypes and penetrance of the variants annotated as pathogenic are unknown, it is hard to know for certain whether they are in fact pathogenic. We opted to report these findings in the hope that they may be valuable for researchers in this area.

To further characterize the variants we have identified among the 14 Xavante samples, we looked for possible SNP-traits associations based on NHGRI genome-wide association studies (GWAS) catalog^27^. Over the past decades, more than 4000 GWAS studies have been published, reporting over 200,000 associations to different phenotypes. We used the most recent version of the GWAS catalog which was mapped to GRCh38.p13, with a significance cutoff of 10^–5^. A total of 22,903 variants were present in at least one of the 14 Xavante exomes and 973 variants were shared by all 14 Xavante samples. We then looked at significant SNP-trait associated variants among these 973 SNPs which also have different frequencies from the 1000 Genomes reference populations (Supplementary Table 9). Most of these variants have been previously associated with human morphology, lipid metabolism and blood cell function. This certainly looks intriguing, and might be useful for those who are interested in validating the association within this population.

## Discussion

The carcinogenic process involves a multiplicity of mechanisms consisting of both somatic and germinal pathogenic or susceptibility variants, whose balance and complexity in each type of cancer are different.

The vast majority of breast cancer is sporadic, representing on average 90% of all cases. Cancer-causing genetic changes can be inherited^28^, but can also be acquired and accumulated during one’s lifetime, as a result of errors that occur as cells divide (1 × 10^−10^ mutations/bp/cell division), or from exposure to environmental carcinogenic substances that damage DNA^29^. The International Agency for Research on Cancer (IARC) of WHO has identified and classified more than 500 agents representing potential threats for human beings as carcinogens. Some 88% of those potential agents classified by IARC are DNA-damaging agents, i.e. they carry an unmistakable mutagenic or clastogenic activity. However, aside from somatic mutations, we still face a scarcity of data on the role the mutational germinal load has on the carcinogenesis process^30^. By analyzing a population of Xavante with no observed incidence of breast cancer, we explored if and how the low mutation germline load could play a role in this pathological process when compared to populations with high mutational load and high breast cancer incidence.

The study of Xavante exomes provides useful insight into the role of germline mutation germinal load in these populations, indicating that their low germinal mutation load may explain, at least in part, the absence of breast cancer in Xavante women. Among all 1000 Genomes control populations, the African populations seems to have more mutated genes overall than all other normal populations which is also observed by Auton et al.^31^. The impact of the higher mutational load in African populations in the context of breast cancer warrants further investigation and studies. Breast cancer is a complicated and multi-factor disease with different subtypes, and disease risk and mechanism vary in different ethnic groups. One major research area in assessing breast cancer risk is in the heritability of the disease. But known risk genes (*BRCA1/BRCA2* and others) are insufficient to explain all inherited breast cancer risk. This is why more recent studies have pursued polygenic risk assessment^32^ in order to acquire a better estimates. We have shown in our study that the genomic signatures of the Xavante cohort appear to be rather unique. We believe that both the genetic distinctiveness of this population and low germinal mutational load are important in giving them the protection against breast cancer.

The demographic and adaptive processes experienced by this indigenous group may have shaped their genetic architecture, and this might have important implications in biological and health related processes of the population^33^. This population has an overall low germline mutation load when compared to other populations, which could be the result of its unique environmental, cultural, demographic and genetic histories. The specific genomic profile of the Xavante could indicate that lifestyle and environmental factors played an important role in shaping the genetic structure of this population and might have been the reason for the observed absence of mutations^34^.

Polygenic inheritance, involving many common genetic variants of moderate to low penetrance, plays a greater role than rare monogenic mutations^18^. Polygenic risk score (PRS) is an estimate of an individual’s genetic liability to a trait or disease, calculated according to their genotype profile and relevant genome-wide association study data^35^. It provides a quantitative metric of individual inherited risk based on the cumulative impact of many common polymorphisms. Weights are assigned to each genetic variant according to the strength of their association with disease risk^18^. The Xavante, when compared to breast cancer germline normal control samples, displayed an extremely low polygenic risk score for breast cancer development.

The findings in this study could contribute to understanding the mechanisms of initialization in sporadic breast cancer and promote new approaches for early prevention and detection of the disease. The low number of mutagenic changes seen in the Xavante population may assist in the identification of novel loci of initialization of the process that triggers the changes linked to breast cancer, in addition to one that have already been identified^36^.

Although the number of samples in our study is small, the Xavante constitute a population with extremely low rate of interethnic relationships and has been shown to be genetically uniform^37^. Our study is unique in reporting the possible role played by germinal mutational events in the development of breast cancer. This is the first effort in trying to characterize whole exomes of the Xavante to look for genetic connections to the absence of breast cancer incidences in this population. This work will shed light onto identification of genetic variants associated with biological and clinical traits in this indigenous group.

This study concerns and is centered on female Xavante Indians. Since there are grounds to believe that male breast cancer may display different genetic determinants^38^, the data presented applies solely to female breast cancer.

We also want to emphasize the limitation of our PRS analysis system we adapted in this paper. These specific genome-wide polygenic scores will not give optimal predictive power for other ethnic groups^39^ since the scoring system has been derived from samples of primarily European ancestry. But this is the sample set in which the most genetic studies have been done to date and this scoring system has covered the largest number of genetic markers related to breast cancer risk. We anticipate in the very near future there will be genomic studies expanded to other non-European ethnic groups since the whole biomedical community are more aware of crucial equity issues.

This work does not intend to supply a complete set of data on the genetic mechanisms associated with breast cancer, but to add data on the possible role of germinal mutated genes in breast carcinogenesis. Indeed, it becomes increasingly clear that besides somatic mutations which further evolve during the carcinogenic process leading to oligogenic clones that may determine the prognosis of the tumor and its drug resistance^40^, the germinal mutational load may play a central role in cancer risk and carcinogenesis: since the latter may vary among families and population groups such as the Xavante, this may explain the age-dependence of cancer initiation and thus breast cancer incidence. Indeed, mutation accumulation in somatic cells is a time-dependent process and a stochastic one, adding to mutations inherited as the germinal mutation load. Considering the gold standard number of 7–11 driver mutations in a somatic cell to trigger and sustain a cancer process^41^, one can be led to accept that a germinal mutational load would increase the risk of cancer by adding to mutations acquired during a lifetime due to environmental carcinogens.

The data obtained in the genetic analysis of the Xavante samples in this study reveals that there is significant difference between women of this group and of other ethnicities, showing possible existence of unstudied data to be found in the human genome to be found related to breast cancer. Many other factors could be implicated in the development of breast cancer or in protection against it. Our findings in this study may bring a new insight into human carcinogenesis, highlighting the importance of germinal mutational load beyond environmentally-driven or somatic mutational changes.

More studies in this unique group of people will be needed to further understand their genetic characteristics in relation to breast cancer risk. Furthermore, how other factors, including reproduction history (age at first full term pregnancy for example) and environmental exposures, may also play a role in the differences found and will need to be investigated in order to gain a more complete understanding.

## Supplementary Information

Supplementary Information.

## Data Availability

The data generated during the current study are available in Sequence Read Archive (SRA) with submission ID SUB11354493.
