# Supplementary material for: Genetic determinants and absence of breast cancer in Xavante Indians in Sangradouro Reserve, Brazil
Source: Sci Rep. 2023 Jan 26;13:1452. doi: 10.1038/s41598-023-28461-y (PMC9879933; doi:10.1038/s41598-023-28461-y)
Supplement: Supplementary file 1 — Supplementary Information. [file 41598_2023_28461_MOESM1_ESM.pdf]

Supplemental Information

Supplementary Figure 1: Coverage plot of the 14 Xavante Indian Exomes which indicate the read depth of all Xavante samples across the exonic target region. Agilent SureSelect All Exon V6 target region was used for this calculation. More than half of the target region is covered by more than 100X.

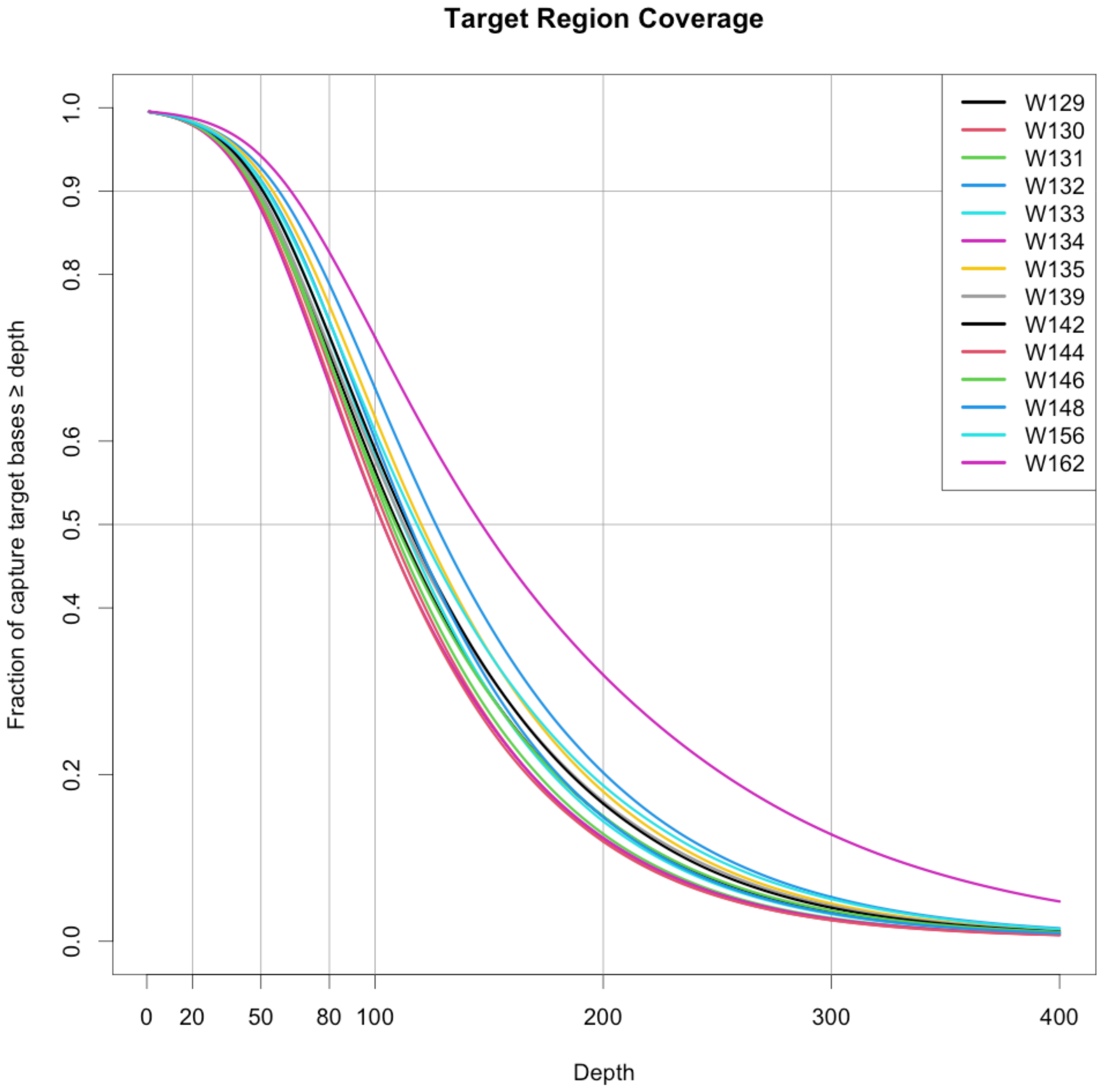

Supplementary Figure 2: Global ancestry proportion analysis of all samples using ADMIXTURE. Results shown are for k=4 to 11 (number of clusters) based on 291,984 SNPs from total of 2,566 samples. The analysis includes 14 Xavante Indians, 522 white, 671 black, 515 East-Asian, 348 Hispanic and 492 South Asian. Each vertical bar represents an individual and the Y-axis represents the proportion of the genome assigned to each of the ancestral clusters as shown on the right side of the plot.

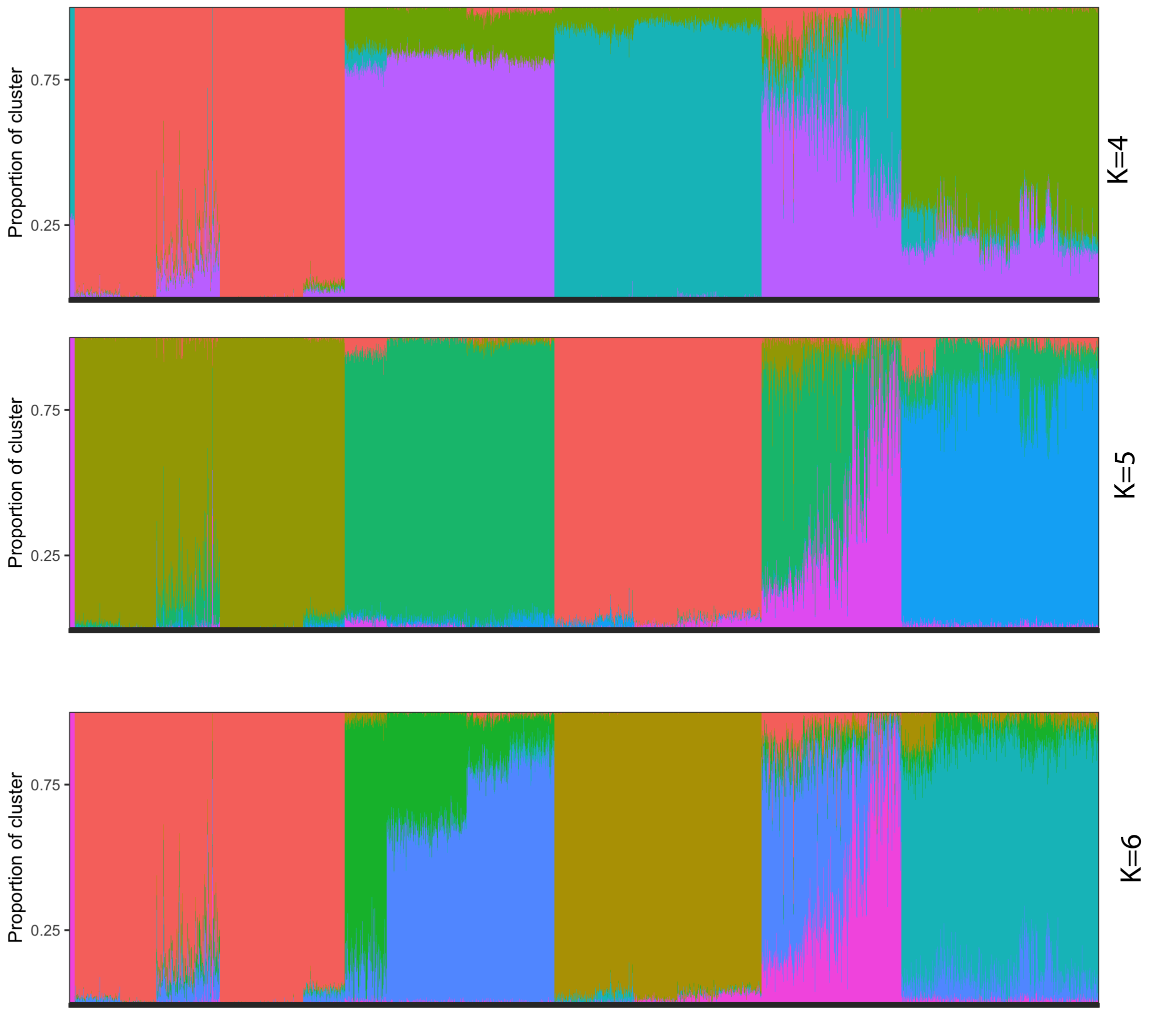

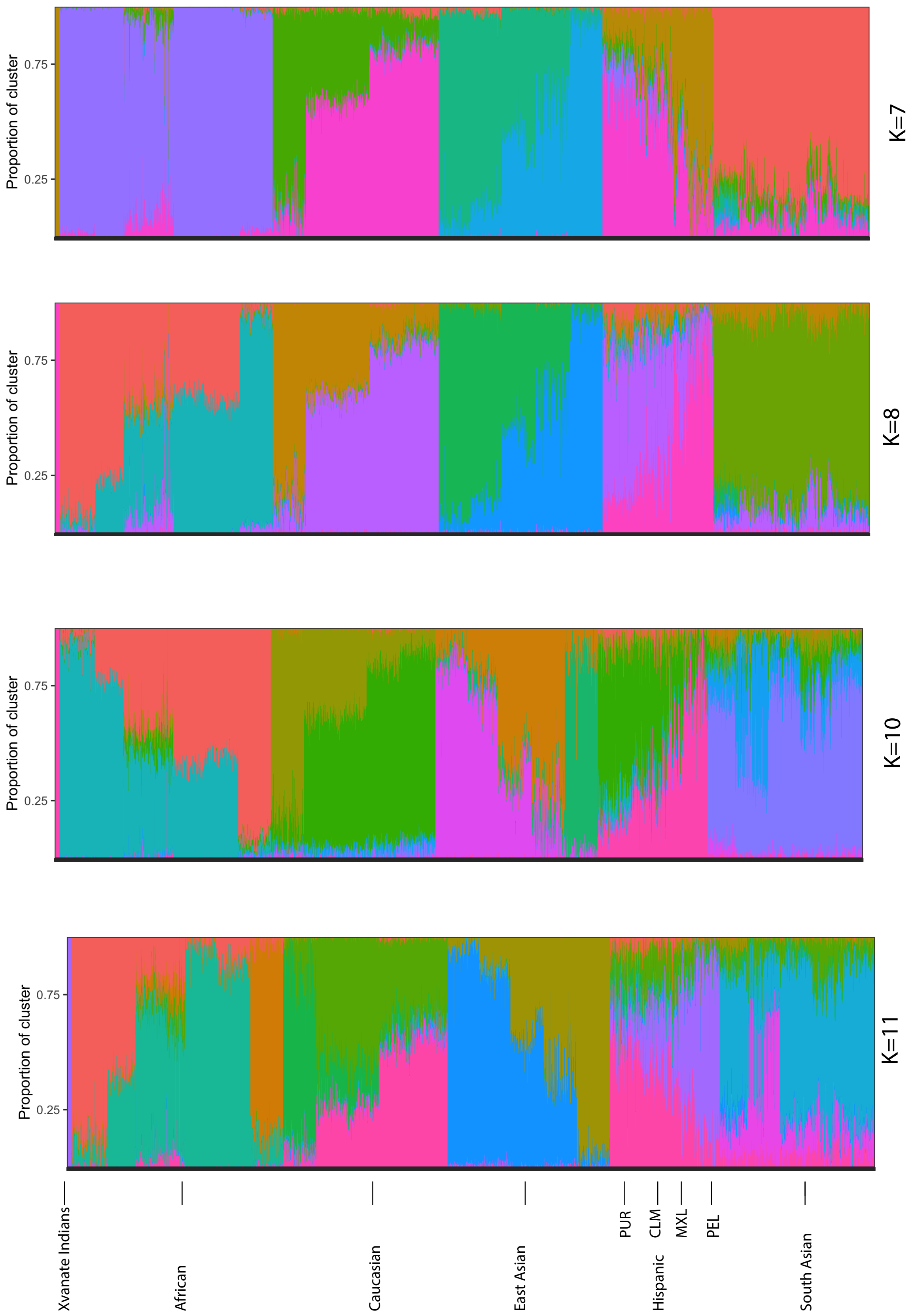
Proportion of cluster
0.75
0.25
K=7
K=8
K=10
K=11
Xvanate Indians
African
Caucasian
East Asian
PUR
CLM
MXL
PEL
Hispanic
South Asian

Supplementary Figure 3. Cross-Validation errors when fitting different values of K using ADMIXTURE. When K=9, it gives the smallest cross validation errors which indicates that 9 clusters would be the best fit.

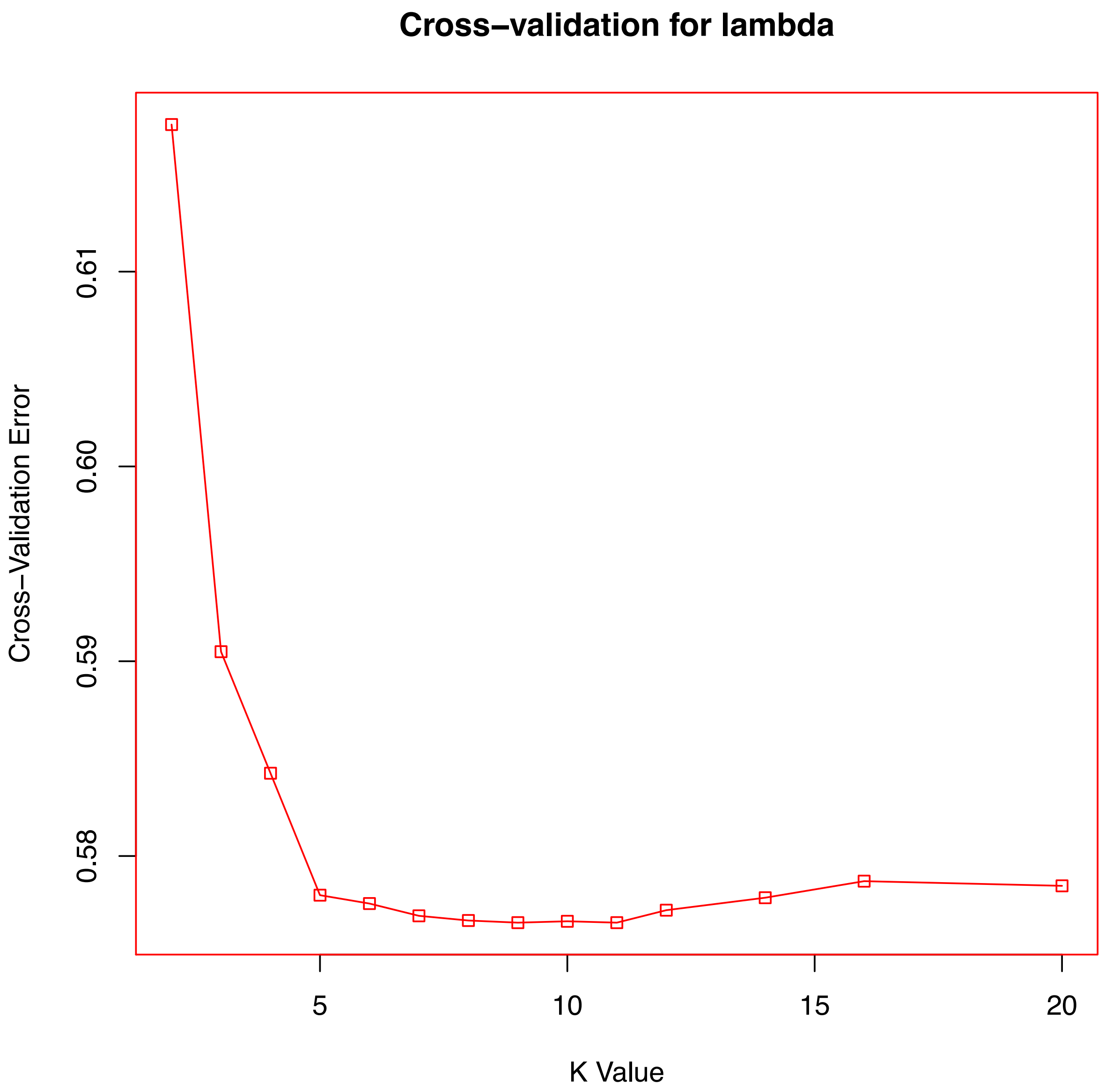

Supplementary Table 1: Demographic information of 14 Xavante Indian Samples Included in this study.

Sample	Age	Weight (Kg)	Height (Cm)	Marital Status	Number Children	Duration Of Breastfeeding (Months)	Age Of Menarche (Years)	Age Of Menopause (Years)	Age at First Childbirth (Years)	Age at Last Childbirth (Years)
W129	18	58	155	Married	2	24	13	N/A	14	16
W130	31	80	167	Married	3	24	Does Not Remember	N/A	18	28
W131	64	64	161	Married	10	18	Does Not Remember	60	20	52
W132	19	64	154	Married	2	12	13	N/A	16	17
W133	14	63	154	Married	1	Ongoing	13	N/A	13	N/A
W134	16	82	156	Married	0	0	Does Not Remember	N/A	N/A	N/A
W135	14	64	154	Single	0	0	12	N/A	N/A	N/A
W139	35	74	166	Married	8	18	Does Not Remember	N/A	18	33
W142	14	60	157	Married	1	Ongoing	13	N/A	14	N/A
W144	22	59	153	Married	3	12	13	N/A	15	20
W146	23	71	154	Married	1	12	14	N/A	19	Pregnant
W148	16	70	162	Married	1	12	12	N/A	15	N/A
W156	64	66	155	Married	7	12	14	55	15	42
W162	39	76	158	Married	6	24	Does Not Remember	N/A	17	29

Supplementary Table 2: Sequence Coverage Depth for Each Sequenced Sample

Sample	>=5X	10x	30x *	50x	100x
W129	0.993	0.990	0.964	0.896	0.565
W130	0.993	0.990	0.961	0.890	0.540
W131	0.993	0.990	0.962	0.894	0.550
W132	0.993	0.991	0.972	0.928	0.663
W133	0.993	0.990	0.967	0.906	0.582
W134	0.993	0.990	0.959	0.878	0.523
W135	0.994	0.991	0.971	0.920	0.629
W139	0.993	0.990	0.964	0.896	0.577
W142	0.993	0.990	0.966	0.903	0.590
W144	0.993	0.990	0.961	0.883	0.525
W146	0.993	0.990	0.962	0.889	0.557
W148	0.993	0.990	0.968	0.913	0.602
W156	0.993	0.991	0.969	0.912	0.612
W162	0.999	0.997	0.983	0.946	0.728

* At least 30X coverage for more than 95% of the exome.

Supplemental Table 3: Number of SNVs (Single Nucleotide Variants)identified by ExomeSeq for each sample includes number of SNVs total, SNVs located in coding region, missense SNVs and NonSense SNVs

	SNV		Coding		Missense		NonSense	
Sample	Total	Novel	Total	Novel	Total	Novel	Total	Novel
W129	370738	26859(7.2)	19653	278(1.4)	7766	110(1.4)	52	2(3.8)
W130	333354	23760(7.1)	19834	259(1.3)	7949	102(1.3)	56	2(3.6)
W131	333315	18117(5.4)	19473	232(1.2)	7810	103(1.3)	52	3(5.8)
W132	361259	20353(5.6)	19735	259(1.3)	7850	113(1.4)	59	4(6.8)
W133	346856	18167(5.2)	19674	263(1.3)	7801	111(1.4)	59	4(6.8)
W134	332508	23770(7.1)	19561	281(1.4)	7839	107(1.4)	60	3(5)
W135	384363	25212(6.6)	19737	263(1.3)	7751	107(1.4)	56	1(1.8)
W139	332885	17027(5.1)	19570	230(1.2)	7778	99(1.3)	58	2(3.4)
W142	352051	22682(6.4)	19830	258(1.3)	7931	105(1.3)	60	4(6.7)
W144	335222	21868(6.5)	19865	252(1.3)	7962	108(1.4)	60	3(5)
W146	530740	46438(8.7)	19937	343(1.7)	7679	117(1.5)	64	4(6.2)
W148	370778	32319(8.7)	19676	308(1.6)	7856	124(1.6)	61	4(6.6)
W156	358527	17002(4.7)	19619	250(1.3)	7750	97(1.3)	58	3(5.2)
W162	413737	20618(5)	19778	249(1.3)	7738	116(1.5)	48	3(6.2)

Supplementary Table 4: Homozygous and heterozygous SNVs identified by ExomeSeq for each sample.

Sample	SNV_total	Homozygous (%)	Heterozygous (%)*
W129	370738	286493(77.3)	84245(22.7)
W130	333354	248528(74.6)	84826(25.4)
W131	333315	253376(76)	79939(24)
W132	361259	273730(75.8)	87529(24.2)
W133	346856	262276(75.6)	84580(24.4)
W134	332508	250318(75.3)	82190(24.7)
W135	384363	292313(76.1)	92050(23.9)
W139	332885	248148(74.5)	84737(25.5)
W142	352051	263243(74.8)	88808(25.2)
W144	335222	250168(74.6)	85054(25.4)
W146	530740	426044(80.3)	104696(19.7)
W148	370778	280990(75.8)	89788(24.2)
W156	358527	272105(75.9)	86422(24.1)
W162	413737	313593(75.8)	100144(24.2)

* Heterozygous SNVs is only about 25% in all samples

Supplemental Table 5: Number of Insertions (INS) and Deletions (DEL) Identified by ExomeSeq for Each Sample

Sample	INS	Novel (%)*	DEL	Novel (%)*
W129	22080	1752(7.9)	28009	4090(14.6)
W130	19336	1448(7.5)	24890	3511(14.1)
W131	20370	1539(7.6)	26060	3855(14.8)
W132	21926	1745(8)	28156	4071(14.5)
W133	21437	1587(7.4)	27186	3556(13.1)
W134	19242	1417(7.4)	24206	3259(13.5)
W135	23516	1804(7.7)	30106	4317(14.3)
W139	20401	1495(7.3)	25518	3489(13.7)
W142	20851	1563(7.5)	26901	3736(13.9)
W144	20103	1488(7.4)	25035	3340(13.3)
W146	29990	2295(7.7)	38336	5538(14.4)
W148	22020	1741(7.9)	28414	4146(14.6)
W156	22459	1743(7.8)	28617	3981(13.9)
W162	26544	2213(8.3)	34547	5078(14.7)

*Novel insertions are at average of 7% and novel deletions are at average of 14%.

Supplemental Table 6: Average number of mutated genes of Xavante Indian samples, the 1000 Genome Project normal population and TCGA breast normal control samples, as well as the P values when all population were compared with Xavante Indian samples.

Population	P value when compare to Xavante	Mean	Standard Deviation
Xavante		268.93*	13.74
ACB	1.49E-08	910.41	42.46
ASW	6.24E-08	844.60	75.55
ESN	1.74E-08	940.85	22.57
GWD	7.85E-09	937.16	35.42
LWK	8.96E-09	998.02	38.67
MSL	1.75E-08	991.87	40.02
YRI	8.95E-09	929.59	30.03
CLM	1.18E-08	595.58	47.24
MXL	9.49E-08	552.56	28.51
PEL	2.30E-08	547.52	44.72
PUR	1.38E-08	600.48	57.59
CDX	1.02E-08	631.00	32.30
CHB	7.46E-09	628.31	40.29
CHS	1.10E-08	617.68	29.92
JPT	1.61E-08	644.25	20.43
KHV	1.10E-08	636.53	24.77
CEU	1.37E-08	534.26	25.26
FIN	5.35E-09	515.23	22.60
GBR	1.27E-08	521.57	32.11
IBS	1.10E-08	553.51	23.55
TSI	1.02E-08	568.59	25.67
BEB	2.29E-08	658.59	35.52
GIH	1.61E-08	643.65	24.97
ITU	2.53E-08	652.49	35.38
PJL	1.61E-08	639.00	30.25
STU	1.75E-08	655.26	25.62
Lobular Breast	1.59E-09	669.27	247.32
Ductal Breast	1.51E-09	727.79	201.68

*Our cohort, the Xavante Indians, is having the lowest mutation among all populations.

Supplemental Table 7: Average number of mutated genes known to be related to breast cancer risk in Xavante Indian samples, the 1000 Genome Project normal population and TCGA breast normal control samples, as well as the P values when all population were compared with Xavante Indian samples.

Population	P value when compare to Xavante	Mean	Standard Deviation
Xavante		0.14*	0.36
ACB	0.05	0.65	0.93
ASW	0.09	0.57	0.81
ESN	0.02	0.62	0.74
GWD	0.00	0.90	0.91
LWK	0.03	0.75	1.01
MSL	0.00	0.98	0.94
YRI	0.01	0.79	0.97
CLM	0.91	0.17	0.43
MXL	0.92	0.16	0.37
PEL	0.99	0.16	0.43
PUR	0.61	0.24	0.52
CDX	0.36	0.28	0.49
CHB	0.17	0.39	0.62
CHS	0.40	0.28	0.53
JPT	0.25	0.33	0.56
KHV	0.34	0.28	0.50
CEU	0.74	0.20	0.45
FIN	0.72	0.18	0.39
GBR	0.39	0.25	0.44
IBS	0.40	0.28	0.53
TSI	0.93	0.15	0.41
BEB	0.30	0.36	0.69
GIH	0.80	0.21	0.50
ITU	0.30	0.30	0.51
PJL	0.58	0.23	0.47
STU	0.15	0.38	0.57
Lobular Breast	8.24E-09	1.60	0.88
Ductal Breast	4.45E-09	1.69	0.77

*Our cohort, the Xavante Indians, is having the lowest mutation in breast cancer risk genes among all populations.

Supplemental Table 8: Rare Variant Mutations Detected within 56 Genes Recommend by ACMG for Incidental Findings.

Variant	Gene	W129	W130	W131	W132	W133	W134	W135	W139	W142	W144	W146	W148	W156	W162	Total
11:47333924.MYBPC3.G->C	MYBPC3	P	.	P	.	.	P	.	.	.	P	P	.	.	.	5
6:7541927.DSP.C->G	DSP	.	.	.	.	.	B	.	B	.	.	.	.	.	.	2
3:123700718.MYLK.T->C	MYLK	U	.	.	.	.	.	.	.	.	.	.	.	.	.	1
1:237785996.RYR2.G->A	RYR2	.	.	.	U	.	.	.	.	.	.	.	.	.	.	1
2:47403214.MSH2.C->T	MSH2	.	.	.	.	.	B	.	.	.	.	.	.	.	.	1
1:45330550.MUTYH.C->T	MUTYH	.	.	.	.	.	.	.	.	U	.	.	.	.	.	1
3:38550832.SCN5A.C->T	SCN5A	.	.	.	.	.	.	.	.	.	.	.	U	.	.	1
13:32379413.BRCA2.G->A	BRCA2	.	.	.	.	.	.	.	.	.	.	.	B	.	.	1

*Clin Var annotation. P: pathogenic; B: benign. U: uncertain or unknown.

Supplemental Table 9: Variants shared by all 14 Xavante Indians samples which have significant SNP-trait association, and also have different population frequencies when compared to 1000 Genome AFR,EAS and EUR populations (population frequencies are shown in column 6-9).

Chr	Pos	Gene	SNPS	MAPPED_TRAIT	Xavante	AFR	EAS	EUR
11	3228599	AC109309.1, MRGPRE	rs11026040	Abnormality of chromosome segregation	1	0.28	0.13	0.14
14	69236441	EXD2	rs3211166	Abnormality of refraction	1	0.06	0.91	0.29
17	64506317	DDX5	rs1991401	Abnormality of refraction	1	0.02	0.41	0.36
9	136364010	CARD9	rs1135314	acute insulin response measurement	1	0.23	0.04	0.33
11	61779120	MYRF, TMEM258	rs174530	acylcarnitine measurement	1	0.03	0.57	0.38
17	76651894	ST6GALNAC 1 - RNU6-227P	rs9889862	adolescent idiopathic scoliosis	1	0.30	0.62	0.35
14	22168978	TRAV30	rs11157436	adolescent idiopathic scoliosis	1	0.06	0.61	0.19
11	61783884	MYRF, TMEM258	rs174535	adult onset asthma	1	0.15	0.57	0.35
9	7174673	KDM4C	rs913588	age at menarche	1	0.32	0.12	0.48
21	39232503	BRWD1	rs2836950	age at menarche	1	0.20	0.23	0.40
2	27492549	FNDC4	rs2303369	age at menopause	1	0.37	0.17	0.37
1	161215268	FCER1G	rs2070901	age at onset, asthma	1	0.43	0.45	0.27
19	7152692	INSR	rs3745548	age at onset, Myopia	1	0.00	0.49	0.01
1	161215268	FCER1G	rs2070901	aggressive periodontitis	1	0.43	0.45	0.27
11	61803876	FADS1, FADS2	rs174548	albumin:globulin ratio measurement	1	0.18	0.55	0.31
15	45428399	SPATA5L1 - C15orf48	rs16943246	albuminuria	1	0.27	0.72	0.28
11	113789854	ATF4P4, AP003170.2	rs1713676	alcohol consumption measurement	1	0.16	0.42	0.56
1	161215268	FCER1G	rs2070901	allergic rhinitis	1	0.43	0.45	0.27
22	41265150	RANGAP1	rs2235852	allergy	1	0.21	0.55	0.34
1	161215268	FCER1G	rs2070901	allergy	1	0.43	0.45	0.27
1	161217875	FCER1G	rs2070902	allergy	1	0.41	0.40	0.26
11	61803311	FADS1, FADS2	rs174547	alpha-linolenic acid measurement	1	0.02	0.57	0.35
11	61781087	MYRF, TMEM258	rs509360	alpha-linolenic acid measurement	1	0.08	0.57	0.71
11	20601477	SLC6A5	rs2241941	amino acid measurement	1	0.33	0.36	0.35
1	155957961	ARHGEF2	rs2364403	amyotrophic lateral sclerosis, age at onset	1	0.27	0.78	0.18
11	126175935	AP001893.3	rs7105310	androstenedione measurement, estrogen-receptor positive breast cancer, estrone measurement	1	0.16	0.12	0.11

11	61783884	MYRF, TMEM258	rs174535	ankylosing spondylitis, psoriasis, ulcerative colitis, Crohn's disease, sclerosing cholangitis	1	0.15	0.57	0.35
20	1671323	SIRPB3P	rs3818168	anti-meningococcal C serum bactericidal antibody measurement, response to vaccine	1	0.06	0.18	0.22
22	40963782	RBX1	rs2413631	anxiety	1	0.02	0.03	0.25
14	74911482	RPS6KL1	rs3213716	anxiety	1	0.02	0.58	0.41
6	160716958	PLG	rs783147	apolipoprotein A 1 measurement	1	0.18	0.52	0.45
20	35560231	FER1L4	rs224424	apolipoprotein B measurement	1	.	.	.
20	35437976	GDF5	rs143384	appendicular lean mass	1	.	.	.
20	35437976	GDF5	rs143384	appendicular lean mass	1	.	.	.
3	184338313	FAM131A	rs1881973	appendicular lean mass	1	0.47	0.39	0.38
11	61776027	MYRF, TMEM258	rs174528	arachidonic acid measurement	1	0.45	0.57	0.39
17	39965740	GSDMA	rs3894194	asthma	1	0.26	0.54	0.45
1	161215268	FCER1G	rs2070901	asthma	1	0.43	0.45	0.27
1	161215268	FCER1G	rs2070901	asthma	1	0.43	0.45	0.27
17	64506317	DDX5	rs1991401	asthma	1	0.02	0.41	0.36
1	161215268	FCER1G	rs2070901	asthma	1	0.43	0.45	0.27
1	161215268	FCER1G	rs2070901	asthma	1	0.43	0.45	0.27
11	61783884	MYRF, TMEM258	rs174535	asthma	1	0.15	0.57	0.35
17	39972512	GSDMA	rs8077456	asthma	1	0.24	0.40	0.34
1	161198214	NDUFS2, ADAMTS4	rs33941127	asthma	1	0.41	0.45	0.21
1	161215268	FCER1G	rs2070901	atopic asthma	1	0.43	0.45	0.27
8	142680513	PSCA, JRK	rs2294008	atrophic gastritis	1	0.37	0.34	0.45
4	186082920	TLR3	rs3775291	autoimmune thyroid disease	1	0.03	0.33	0.32
1	113886839	BCL2L15, AP4B1-AS1	rs2358994	autoimmune thyroid disease, type I diabetes mellitus	1	0.02	0.60	0.19
19	51225221	CD33	rs12459419	basophil count	1	0.05	0.19	0.31
12	6390367	LTBR	rs2286599	basophil count	1	0.07	0.03	0.16
19	51225221	CD33	rs12459419	basophil count	1	0.05	0.19	0.31
7	75813412	CCL24	rs2302006	basophil count, eosinophil count	1	0.27	0.56	0.18
3	169769713	AC078802.1	rs9811216	basophil count, eosinophil count	1	0.30	0.68	0.26
11	61803876	FADS1, FADS2	rs174548	basophil count, eosinophil count	1	0.18	0.55	0.31
16	31140200	PRSS36	rs8047469	bipolar disorder	1	.	.	.
11	66715794	SPTBN2	rs12805133	bipolar disorder	1	0.24	0.43	0.45
16	31140200	PRSS36	rs8047469	bipolar I disorder	1	.	.	.

6	166938616	AL159163.1, RNASET2	rs3777722	birth measurement, spontaneous preterm birth	1	0.03	0.41	0.11
11	2135306	INS-IGF2, IGF2, AC132217.2	rs3213225	birth weight	1	0.40	0.29	0.58
8	142680513	PSCA, JRK	rs2294008	bladder carcinoma	1	0.37	0.34	0.45
8	142680513	PSCA, JRK	rs2294008	bladder carcinoma	1	0.37	0.34	0.45
11	61803876	FADS1, FADS2	rs174548	blood metabolite measurement	1	0.18	0.55	0.31
11	61783884	MYRF, TMEM258	rs174535	blood metabolite measurement	1	0.15	0.57	0.35
11	61803876	FADS1, FADS2	rs174548	blood metabolite measurement	1	0.18	0.55	0.31
11	61783884	MYRF, TMEM258	rs174535	blood metabolite measurement	1	0.15	0.57	0.35
11	61813163	FADS1, FADS2	rs174556	blood metabolite measurement	1	0.02	0.55	0.30
11	61792609	TMEM258	rs174538	blood metabolite measurement	1	0.02	0.56	0.31
5	148476770	HTR4	rs7733088	blood pressure, chronic obstructive pulmonary disease	1	0.29	0.64	0.37
17	7651454	ATP1B2	rs1642762	blood protein measurement	1	0.06	0.49	0.57
19	18599725	CRLF1	rs2238647	blood protein measurement	1	0.08	0.53	0.23
3	141443286	ZBTB38	rs62282002	blood protein measurement	1	0.01	0.22	0.09
11	61779120	MYRF, TMEM258	rs174530	blood protein measurement	1	0.03	0.57	0.38
19	51225221	CD33	rs12459419	blood protein measurement	1	0.05	0.19	0.31
19	10291455	ICAM5	rs901886	blood protein measurement	1	0.09	0.32	0.52
1	147652507	ACP6	rs2153463	blood protein measurement	1	.	.	.
9	136971795	LINC02692	rs11790360	blood protein measurement	1	0.21	0.18	0.53
8	41309119	SFRP1	rs3055861	blood protein measurement	1	0.04	0.40	0.40
10	89009208	FAS	rs7911226	blood protein measurement	1	0.24	0.46	0.31
10	89009208	FAS	rs7911226	blood protein measurement	1	0.24	0.46	0.31
19	10291455	ICAM5	rs901886	blood protein measurement	1	0.09	0.32	0.52
19	10291455	ICAM5	rs901886	blood protein measurement	1	0.09	0.32	0.52
1	203186870	CHI3L1 - CHIT1	rs10399805	blood protein measurement	1	0.44	0.29	0.13
19	10285007	ICAM1, AC011511.2	rs5498	blood protein measurement	1	0.14	0.28	0.47
9	136971795	LINC02692	rs11790360	blood protein measurement	1	0.21	0.18	0.53
9	136971795	LINC02692	rs11790360	blood protein measurement	1	0.21	0.18	0.53
9	136971795	LINC02692	rs11790360	blood protein measurement	1	0.21	0.18	0.53
9	136971795	LINC02692	rs11790360	blood protein measurement	1	0.21	0.18	0.53
6	160716958	PLG	rs783147	blood protein measurement	1	0.18	0.52	0.45
15	53615751	WDR72	rs17730281	blood urea nitrogen measurement	1	0.12	0.49	0.20

12	121012362	C12orf43	rs2264750	blood urea nitrogen measurement	1	0.11	0.47	0.32
20	35437976	GDF5	rs143384	BMI-adjusted hip circumference	1	.	.	.
20	35437976	GDF5	rs143384	BMI-adjusted hip circumference	1	.	.	.
20	35437976	GDF5	rs143384	BMI-adjusted hip circumference	1	.	.	.
20	35437976	GDF5	rs143384	BMI-adjusted hip circumference	1	.	.	.
20	35434589	GDF5-AS1, GDF5	rs224331	BMI-adjusted waist circumference	1	.	.	.
20	35437976	GDF5	rs143384	BMI-adjusted waist-hip ratio	1	.	.	.
20	35434589	GDF5-AS1, GDF5	rs224331	BMI-adjusted waist-hip ratio	1	.	.	.
20	35434589	GDF5-AS1, GDF5	rs224331	BMI-adjusted waist-hip ratio	1	.	.	.
20	35434589	GDF5-AS1, GDF5	rs224331	BMI-adjusted waist-hip ratio	1	.	.	.
20	35434589	GDF5-AS1, GDF5	rs224331	BMI-adjusted waist-hip ratio	1	.	.	.
17	41811190	P3H4	rs13412	BMI-adjusted waist-hip ratio	1	0.23	0.45	0.35
20	35434589	GDF5-AS1, GDF5	rs224331	BMI-adjusted waist-hip ratio	1	.	.	.
20	35437976	GDF5	rs143384	body fat distribution	1	.	.	.
20	35437976	GDF5	rs143384	body fat distribution	1	.	.	.
20	35437976	GDF5	rs143384	body fat distribution	1	.	.	.
20	35437976	GDF5	rs143384	body fat distribution	1	.	.	.
20	35437976	GDF5	rs143384	body fat distribution	1	.	.	.
6	5260703	LYRM4	rs2224391	body height	1	0.84	0.26	0.25
20	35437976	GDF5	rs143384	body height	1	.	.	.
6	25916751	SLC17A2	rs1865760	body height	1	0.14	0.70	0.40
11	61803311	FADS1, FADS2	rs174547	body height	1	0.02	0.57	0.35
20	35437976	GDF5	rs143384	body height	1	.	.	.
6	26157253	H1-4 - H2BC5	rs4141885	body height	1	0.02	0.65	0.12
20	35437976	GDF5	rs143384	body height	1	.	.	.
6	26183874	H2BC6	rs7766641	body height	1	0.02	0.76	0.28
6	26183874	H2BC6	rs7766641	body height	1	0.02	0.76	0.28
9	130596504	FUBP3	rs10901216	body height	1	0.05	0.49	0.38
7	135672728	STMP1	rs3110823	body height	1	0.23	0.50	0.15
5	141184688	AC244517.6, PCDHB16, AC244517.4, AC244517.11	rs17844666	body height	1	0.05	0.53	0.23
11	2165105	TH	rs2070762	body height	1	0.20	0.47	0.48
1	36307804	SH3D21	rs35343437	body height	1	0.12	0.72	0.44

16	89637957	DPEP1	rs1126464	body height	1	0.08	0.36	0.25
20	35437976	GDF5	rs143384	body height	1	.	.	.
15	40032280	EIF2AK4	rs4432245	body mass index	1	0.28	0.46	0.05
1	19608406	AL031727.1, MICOS10-NBL1, MICOS10, NBL1	rs61740466	body mass index	1	0.01	0.52	0.23
1	19608406	AL031727.1, MICOS10-NBL1, MICOS10, NBL1	rs61740466	body mass index	1	0.01	0.52	0.23
3	154306376	DHX36	rs355754	body mass index	1	0.39	0.49	0.42
22	40273644	TNRC6B	rs733381	body mass index	1	0.06	0.29	0.20
12	107319919	BTBD11	rs111260184	body mass index	1	0.22	0.41	0.49
12	123007565	PITPNM2	rs3897102	body mass index	1	0.03	0.66	0.43
12	107319919	BTBD11	rs111260184	body mass index	1	0.22	0.41	0.49
6	51933962	PKHD1	rs1884953	body mass index	1	0.09	0.17	0.18
3	154301098	DHX36	rs9438	body mass index	1	0.39	0.48	0.41
20	35437976	GDF5	rs143384	body weight	1	.	.	.
20	35437976	GDF5	rs143384	body weights and measures	1	.	.	.
20	35437976	GDF5	rs143384	body weights and measures, body height	1	.	.	.
9	130596504	FUBP3	rs10901216	bone density	1	0.05	0.49	0.38
9	130596504	FUBP3	rs10901216	bone density	1	0.05	0.49	0.38
12	101293265	UTP20	rs2290720	brain measurement, hippocampal volume	1	0.12	0.40	0.39
20	62547989	MIR1-1HG-AS1	rs12479469	brain stem volume measurement	1	0.32	0.47	0.33
20	62547989	MIR1-1HG-AS1	rs12479469	brain volume measurement	1	0.32	0.47	0.33
20	62547989	MIR1-1HG-AS1	rs12479469	brain volume measurement	1	0.32	0.47	0.33
20	62547989	MIR1-1HG-AS1	rs12479469	brain volume measurement	1	0.32	0.47	0.33
20	35437976	GDF5	rs143384	brain volume measurement	1	.	.	.
6	126643364	AL356534.1	rs4273712	brain volume measurement, intracranial volume measurement	1	0.05	0.44	0.28
1	113905767	DCLRE1B	rs11552449	breast carcinoma	1	0.02	0.59	0.19
1	113905767	DCLRE1B	rs11552449	breast carcinoma	1	0.02	0.59	0.19
1	113905767	DCLRE1B	rs11552449	breast carcinoma	1	0.02	0.59	0.19
9	136395373	SNAPC4	rs4266763	C-reactive protein measurement	1	0.36	0.32	0.39
15	58431280	LIPC, ALDH1A2	rs1077834	C-reactive protein measurement, high density	1	0.57	0.42	0.21

				lipoprotein cholesterol measurement				
11	61802358	FADS2, FADS1	rs174546	C-reactive protein measurement, high density lipoprotein cholesterol measurement	1	0.02	0.57	0.35
4	87834676	MEPE	rs7698623	cardiovascular disease	1	.	.	.
10	69572448	NEUROG3	rs4536103	carotid artery intima media thickness	1	0.26	0.31	0.67
3	57102557	IL17RD	rs17057718	cerebrospinal fluid biomarker measurement	1	0.04	0.38	0.15
17	39965740	GSDMA	rs3894194	childhood onset asthma	1	0.26	0.54	0.45
1	161215268	FCER1G	rs2070901	childhood onset asthma	1	0.43	0.45	0.27
1	161215268	FCER1G	rs2070901	childhood onset asthma	1	0.43	0.45	0.27
11	61803876	FADS1, FADS2	rs174548	cholesteryl ester 16:0 measurement	1	0.18	0.55	0.31
11	61783884	MYRF, TMEM258	rs174535	cholesteryl ester 18:3 measurement	1	0.15	0.57	0.35
11	61803311	FADS1, FADS2	rs174547	cholesteryl ester 20:4 measurement	1	0.02	0.57	0.35
11	61803876	FADS1, FADS2	rs174548	cholesteryl ester 20:5 measurement	1	0.18	0.55	0.31
15	53615751	WDR72	rs17730281	chronic kidney disease	1	0.12	0.49	0.20
11	61790354	TMEM258	rs102274	chronic kidney disease, serum metabolite measurement	1	0.03	0.57	0.35
11	61826344	FADS2, FADS1	rs174568	chronic kidney disease, serum metabolite measurement	1	0.02	0.57	0.35
11	61803876	FADS1, FADS2	rs174548	chronic kidney disease, serum metabolite measurement	1	0.18	0.55	0.31
11	61803876	FADS1, FADS2	rs174548	chronic kidney disease, serum metabolite measurement	1	0.18	0.55	0.31
11	61803876	FADS1, FADS2	rs174548	chronic kidney disease, serum metabolite measurement	1	0.18	0.55	0.31
11	61811991	FADS1, FADS2	rs174554	chronic kidney disease, serum metabolite measurement	1	0.02	0.57	0.34
11	61803876	FADS1, FADS2	rs174548	chronic kidney disease, serum metabolite measurement	1	0.18	0.55	0.31
11	61803876	FADS1, FADS2	rs174548	chronic kidney disease, serum metabolite measurement	1	0.18	0.55	0.31
11	61803876	FADS1, FADS2	rs174548	chronic kidney disease, serum metabolite measurement	1	0.18	0.55	0.31
11	61803876	FADS1, FADS2	rs174548	chronic kidney disease, serum metabolite measurement	1	0.18	0.55	0.31
11	61826344	FADS2, FADS1	rs174568	chronic kidney disease, serum metabolite measurement	1	0.02	0.57	0.35
11	61826344	FADS2, FADS1	rs174568	chronic kidney disease, serum metabolite measurement	1	0.02	0.57	0.35
11	61776027	MYRF, TMEM258	rs174528	chronic kidney disease, serum metabolite measurement	1	0.45	0.57	0.39
11	61776027	MYRF, TMEM258	rs174528	chronic kidney disease, serum metabolite measurement	1	0.45	0.57	0.39
11	61776027	MYRF, TMEM258	rs174528	chronic kidney disease, serum metabolite measurement	1	0.45	0.57	0.39
11	61781553	TMEM258, MYRF	rs174533	chronic kidney disease, serum metabolite measurement	1	0.03	0.57	0.35

11	61779120	MYRF, TMEM258	rs174530	chronic kidney disease, serum metabolite measurement	1	0.03	0.57	0.38
11	61776027	MYRF, TMEM258	rs174528	chronic kidney disease, serum metabolite measurement	1	0.45	0.57	0.39
5	35037010	AGXT2	rs37369	chronic kidney disease, urinary metabolite measurement	1	0.63	0.57	0.09
5	35037010	AGXT2	rs37369	chronic kidney disease, urinary metabolite measurement	1	0.63	0.57	0.09
5	35037010	AGXT2	rs37369	chronic kidney disease, urinary metabolite measurement	1	0.63	0.57	0.09
5	35037010	AGXT2	rs37369	chronic kidney disease, urinary metabolite measurement	1	0.63	0.57	0.09
5	35037010	AGXT2	rs37369	chronic kidney disease, urinary metabolite measurement	1	0.63	0.57	0.09
5	35037010	AGXT2	rs37369	chronic kidney disease, urinary metabolite measurement	1	0.63	0.57	0.09
5	35037010	AGXT2	rs37369	chronic kidney disease, urinary metabolite measurement	1	0.63	0.57	0.09
5	35037010	AGXT2	rs37369	chronic kidney disease, urinary metabolite measurement	1	0.63	0.57	0.09
5	148476770	HTR4	rs7733088	chronic obstructive pulmonary disease	1	0.29	0.64	0.37
6	26183874	H2BC6	rs7766641	cigarettes per day measurement	1	0.02	0.76	0.28
6	26183874	H2BC6	rs7766641	cigarettes per day measurement	1	0.02	0.76	0.28
11	61802358	FADS2, FADS1	rs174546	cis/trans-18:2 fatty acid measurement, trans fatty acid measurement	1	0.02	0.57	0.35
11	61792609	TMEM258	rs174538	cis/trans-18:2 fatty acid measurement, trans fatty acid measurement	1	0.02	0.56	0.31
11	61776027	MYRF, TMEM258	rs174528	cis/trans-18:2 fatty acid measurement, trans fatty acid measurement	1	0.45	0.57	0.39
11	61779765	MYRF, TMEM258	rs108499	cis/trans-18:2 fatty acid measurement, trans fatty acid measurement	1	0.02	0.56	0.33
11	61781087	MYRF, TMEM258	rs509360	cis/trans-18:2 fatty acid measurement, trans fatty acid measurement	1	0.08	0.57	0.71
11	61803876	FADS1, FADS2	rs174548	cis/trans-18:2 fatty acid measurement, trans fatty acid measurement	1	0.18	0.55	0.31
11	61813163	FADS1, FADS2	rs174556	cis/trans-18:2 fatty acid measurement, trans fatty acid measurement	1	0.02	0.55	0.30
11	61803311	FADS1, FADS2	rs174547	cis/trans-18:2 fatty acid measurement, trans fatty acid measurement	1	0.02	0.57	0.35

11	61783884	MYRF, TMEM258	rs174535	cis/trans-18:2 fatty acid measurement, trans fatty acid measurement	1	0.15	0.57	0.35
11	61812288	FADS1, FADS2	rs174555	cis/trans-18:2 fatty acid measurement, trans fatty acid measurement	1	0.02	0.55	0.30
1	209790735	IRF6	rs2235371	Cleft palate, cleft lip	1	0.00	0.43	0.01
8	94529074	VIRMA	rs957448	Cleft palate, cleft lip	1	0.27	0.49	0.31
12	52952966	KRT18 - EIF4B	rs3741442	Cleft palate, cleft lip	1	0.10	0.53	0.02
1	197101646	ASPM	rs10922162	coagulation factor measurement	1	0.02	0.67	0.17
16	89784625	FANCA	rs16966142	coffee consumption	1	0.11	0.58	0.11
11	61781553	TMEM258, MYRF	rs174533	colorectal cancer, colorectal adenoma	1	0.03	0.57	0.35
11	61803311	FADS1, FADS2	rs174547	comprehensive strength index, muscle measurement	1	0.02	0.57	0.35
6	160540105	LPA	rs3798220	coronary artery disease	1	0.00	0.09	0.01
12	57449928	INHBC	rs2229357	coronary artery disease	1	0.08	0.07	0.19
11	65623846	PCNX3	rs12801636	coronary artery disease	1	0.27	0.43	0.22
11	65623846	PCNX3	rs12801636	coronary artery disease	1	0.27	0.43	0.22
11	65623846	PCNX3	rs12801636	coronary artery disease	1	0.27	0.43	0.22
13	43883789	LACC1	rs3764147	Crohn's disease	1	0.30	0.35	0.23
13	43883789	LACC1	rs3764147	Crohn's disease	1	0.30	0.35	0.23
13	43883789	LACC1	rs3764147	Crohn's disease	1	0.30	0.35	0.23
13	43883789	LACC1	rs3764147	Crohn's disease	1	0.30	0.35	0.23
11	61803876	FADS1, FADS2	rs174548	delta-6 desaturase measurement	1	0.18	0.55	0.31
14	41606749	AL121821.2 - LRFN5	rs1111179	depressive symptom measurement	1	0.21	0.28	0.52
22	40963782	RBX1	rs2413631	depressive symptom measurement	1	0.02	0.03	0.25
20	35437976	GDF5	rs143384	developmental dysplasia of the hip	1	.	.	.
16	89637957	DPEP1	rs1126464	diastolic blood pressure	1	0.08	0.36	0.25
16	89637957	DPEP1	rs1126464	diastolic blood pressure	1	0.08	0.36	0.25
11	65617256	PCNX3	rs12790427	diastolic blood pressure	1	0.26	0.42	0.20
11	1884062	LSP1	rs661348	diastolic blood pressure	1	0.14	0.59	0.43
5	58458178	PLK2	rs1848510	diastolic blood pressure	1	0.26	0.51	0.38
11	61803876	FADS1, FADS2	rs174548	dihomo-gamma-linolenic acid measurement	1	0.18	0.55	0.31
11	61803311	FADS1, FADS2	rs174547	docosahexaenoic acid measurement, metabolic syndrome	1	0.02	0.57	0.35
11	61783884	MYRF, TMEM258	rs174535	docosapentaenoic acid measurement	1	0.15	0.57	0.35
11	61803311	FADS1, FADS2	rs174547	docosapentaenoic acid measurement	1	0.02	0.57	0.35
8	142680513	PSCA, JRK	rs2294008	duodenal ulcer	1	0.37	0.34	0.45

1	161215268	FCER1G	rs2070901	Eczema	1	0.43	0.45	0.27
12	120784753	SPPL3	rs3213567	Eczema	1	0.14	0.44	0.54
19	10293271	ICAM5	rs2569702	Eczema	1	0.04	0.25	0.41
1	161215268	FCER1G	rs2070901	Eczema, allergic rhinitis, asthma	1	0.43	0.45	0.27
11	61783884	MYRF, TMEM258	rs174535	eicosapentaenoic acid measurement	1	0.15	0.57	0.35
11	61792609	TMEM258	rs174538	eicosapentaenoic acid measurement	1	0.02	0.56	0.31
7	75813412	CCL24	rs2302006	eosinophil count	1	0.27	0.56	0.18
3	169769713	AC078802.1	rs9811216	eosinophil count	1	0.30	0.68	0.26
11	61803876	FADS1, FADS2	rs174548	eosinophil count	1	0.18	0.55	0.31
11	61803876	FADS1, FADS2	rs174548	eosinophil count	1	0.18	0.55	0.31
7	75813412	CCL24	rs2302006	eosinophil percentage of granulocytes	1	0.27	0.56	0.18
7	75813412	CCL24	rs2302006	eosinophil percentage of leukocytes	1	0.27	0.56	0.18
11	61781087	MYRF, TMEM258	rs509360	erythrocyte count	1	0.08	0.57	0.71
11	61781553	TMEM258, MYRF	rs174533	erythrocyte count	1	0.03	0.57	0.35
12	52077375	ATG101, AC025259.1	rs11521	erythrocyte count	1	0.07	0.44	0.22
12	132528149	FBRSL1	rs4883565	erythrocyte count	1	0.38	0.52	0.22
11	61803876	FADS1, FADS2	rs174548	esterified cholesterol measurement	1	0.18	0.55	0.31
11	61790354	TMEM258	rs102274	esterified cholesterol measurement	1	0.03	0.57	0.35
12	71132634	TSPAN8	rs2270588	eyelid sagging measurement	1	0.15	0.57	0.43
10	46263871	ANTXRLP1	rs2999422	facial attractiveness measurement	1	0.71	0.11	0.42
12	57224836	NXPH4	rs61736007	fat body mass	1	0.05	0.13	0.26
11	61813163	FADS1, FADS2	rs174556	fatty acid measurement, breast milk measurement, parental genotype effect measurement	1	0.02	0.55	0.30
11	61813163	FADS1, FADS2	rs174556	fatty acid measurement, fetal genotype effect measurement, breast milk measurement	1	0.02	0.55	0.30
11	61783884	MYRF, TMEM258	rs174535	fatty acid measurement, oleic acid measurement	1	0.15	0.57	0.35
20	35437976	GDF5	rs143384	femoral neck size	1	.	.	.
5	148476829	HTR4	rs7715901	FEV/FEC ratio	1	0.29	0.64	0.37
5	148476829	HTR4	rs7715901	forced expiratory volume	1	0.29	0.64	0.37
20	35437976	GDF5	rs143384	forced expiratory volume	1	.	.	.
5	148476829	HTR4	rs7715901	forced expiratory volume	1	0.29	0.64	0.37
15	78580777	CHRNA5	rs569207	forced expiratory volume, response to bronchodilator	1	0.32	0.48	0.24

15	78620601	CHRNA3	rs7170068	forced expiratory volume, response to bronchodilator	1	0.23	0.47	0.23
17	81199662	AC027601.3, CEP131	rs906175	Frontotemporal dementia	1	0.02	0.63	0.45
11	61802358	FADS2, FADS1	rs174546	gamma-linolenic acid measurement	1	0.02	0.57	0.35
8	142680513	PSCA, JRK	rs2294008	gastric adenocarcinoma	1	0.37	0.34	0.45
8	142680513	PSCA, JRK	rs2294008	gastric carcinoma	1	0.37	0.34	0.45
8	142680513	PSCA, JRK	rs2294008	gastric carcinoma	1	0.37	0.34	0.45
8	142680513	PSCA, JRK	rs2294008	gastric carcinoma	1	0.37	0.34	0.45
4	88115201	ABCG2	rs2725261	glomerular filtration rate	1	0.09	0.58	0.35
8	22634630	BIN3	rs7838146	glomerular filtration rate	1	0.37	0.58	0.36
8	17313231	MTMR7	rs10096063	glucuronate measurement	1	0.35	0.26	0.14
11	61802358	FADS2, FADS1	rs174546	glycerophospholipid measurement, blood metabolite measurement	1	0.02	0.57	0.35
11	61802358	FADS2, FADS1	rs174546	glycerophospholipid measurement, blood metabolite measurement	1	0.02	0.57	0.35
11	61803311	FADS1, FADS2	rs174547	glycerophospholipid measurement, blood metabolite measurement	1	0.02	0.57	0.35
11	61803311	FADS1, FADS2	rs174547	glycerophospholipid measurement, blood metabolite measurement	1	0.02	0.57	0.35
11	61803311	FADS1, FADS2	rs174547	glycerophospholipid measurement, blood metabolite measurement	1	0.02	0.57	0.35
11	61803311	FADS1, FADS2	rs174547	glycerophospholipid measurement, blood metabolite measurement	1	0.02	0.57	0.35
11	61803311	FADS1, FADS2	rs174547	glycerophospholipid measurement, blood metabolite measurement	1	0.02	0.57	0.35
11	61803311	FADS1, FADS2	rs174547	glycerophospholipid measurement, blood metabolite measurement	1	0.02	0.57	0.35
11	61803311	FADS1, FADS2	rs174547	glycerophospholipid measurement, blood metabolite measurement	1	0.02	0.57	0.35
11	61812288	FADS1, FADS2	rs174555	glycerophospholipid measurement, blood metabolite measurement	1	0.02	0.55	0.30
11	61776027	MYRF, TMEM258	rs174528	gondoic acid measurement	1	0.45	0.57	0.39
11	61812288	FADS1, FADS2	rs174555	granulocyte count	1	0.02	0.55	0.30
9	136378050	SNAPC4	rs3812565	granulocyte percentage of myeloid white cells	1	0.29	0.19	0.35
11	61813163	FADS1, FADS2	rs174556	granulocyte percentage of myeloid white cells	1	0.02	0.55	0.30
7	100854110	SLC12A9	rs12535629	hair color	1	0.06	0.45	0.26
16	89919746	AC092143.1, MC1R	rs885479	hair color	1	0.01	0.62	0.07

9	136434270	INPP5E	rs7851507	HbA1c measurement	1	0.29	0.20	0.38
7	151064793	SLC4A2	rs2303931	heel bone mineral density	1	0.32	0.39	0.26
11	61776489	TMEM258, MYRF	rs174529	heel bone mineral density	1	0.15	0.57	0.38
11	61781553	TMEM258, MYRF	rs174533	hematocrit	1	0.03	0.57	0.35
8	23566245	SLC25A37	rs10992	hematocrit	1	0.10	0.37	0.66
3	56737223	ARHGEF3	rs3772219	hematocrit	1	0.57	0.42	0.29
12	57449928	INHBC	rs2229357	hematocrit	1	0.08	0.07	0.19
11	61781553	TMEM258, MYRF	rs174533	hemoglobin measurement	1	0.03	0.57	0.35
3	56737223	ARHGEF3	rs3772219	hemoglobin measurement	1	0.57	0.42	0.29
6	109444615	MICAL1	rs2236582	hemoglobin measurement	1	0.19	0.35	0.35
7	91873914	MTERF1	rs10266424	hemoglobin measurement	1	0.19	0.46	0.52
3	56737223	ARHGEF3	rs3772219	hemoglobin measurement	1	0.57	0.42	0.29
11	8730342	DENND2B	rs3794153	hemoglobin measurement	1	0.02	0.35	0.44
6	25914573	SLC17A2	rs2071299	hemoglobin measurement	1	0.14	0.70	0.40
12	57449928	INHBC	rs2229357	hemoglobin measurement	1	0.08	0.07	0.19
11	61781553	TMEM258, MYRF	rs174533	hemoglobin measurement	1	0.03	0.57	0.35
6	25770479	SLC17A4	rs4712970	hemoglobin measurement	1	0.19	0.74	0.29
11	61776027	MYRF, TMEM258	rs174528	hemoglobin measurement	1	0.45	0.57	0.39
3	56737223	ARHGEF3	rs3772219	hemoglobin measurement	1	0.57	0.42	0.29
11	61803311	FADS1, FADS2	rs174547	high density lipoprotein cholesterol measurement	1	0.02	0.57	0.35
15	58431280	LIPC, ALDH1A2	rs1077834	high density lipoprotein cholesterol measurement	1	0.57	0.42	0.21
11	61803876	FADS1, FADS2	rs174548	high density lipoprotein cholesterol measurement	1	0.18	0.55	0.31
15	58431476	ALDH1A2, LIPC	rs1800588	high density lipoprotein cholesterol measurement	1	0.56	0.40	0.21
11	61802358	FADS2, FADS1	rs174546	high density lipoprotein cholesterol measurement	1	0.02	0.57	0.35
15	58431227	ALDH1A2, LIPC	rs1077835	high density lipoprotein cholesterol measurement	1	0.57	0.42	0.21
11	61802358	FADS2, FADS1	rs174546	high density lipoprotein cholesterol measurement	1	0.02	0.57	0.35
11	65623846	PCNX3	rs12801636	high density lipoprotein cholesterol measurement	1	0.27	0.43	0.22
1	206453482	SRGAP2	rs2483058	high density lipoprotein cholesterol measurement	1	.	.	.
11	61776489	TMEM258, MYRF	rs174529	high density lipoprotein cholesterol measurement	1	0.15	0.57	0.38
15	58431227	ALDH1A2, LIPC	rs1077835	high density lipoprotein cholesterol measurement	1	0.57	0.42	0.21
15	58431280	LIPC, ALDH1A2	rs1077834	high density lipoprotein cholesterol measurement	1	0.57	0.42	0.21
11	65623846	PCNX3	rs12801636	high density lipoprotein cholesterol measurement	1	0.27	0.43	0.22

15	58431476	ALDH1A2, LIPC	rs1800588	high density lipoprotein cholesterol measurement	1	0.56	0.40	0.21
11	61802358	FADS2, FADS1	rs174546	high density lipoprotein cholesterol measurement	1	0.02	0.57	0.35
11	61802358	FADS2, FADS1	rs174546	high density lipoprotein cholesterol measurement	1	0.02	0.57	0.35
11	61803311	FADS1, FADS2	rs174547	high density lipoprotein cholesterol measurement	1	0.02	0.57	0.35
11	61803876	FADS1, FADS2	rs174548	high density lipoprotein cholesterol measurement	1	0.18	0.55	0.31
11	61811991	FADS1, FADS2	rs174554	high density lipoprotein cholesterol measurement	1	0.02	0.57	0.34
15	58431476	ALDH1A2, LIPC	rs1800588	high density lipoprotein cholesterol measurement	1	0.56	0.40	0.21
15	58431280	LIPC, ALDH1A2	rs1077834	high density lipoprotein cholesterol measurement	1	0.57	0.42	0.21
15	58431227	ALDH1A2, LIPC	rs1077835	high density lipoprotein cholesterol measurement	1	0.57	0.42	0.21
15	58431280	LIPC, ALDH1A2	rs1077834	high density lipoprotein cholesterol measurement	1	0.57	0.42	0.21
15	58431476	ALDH1A2, LIPC	rs1800588	high density lipoprotein cholesterol measurement	1	0.56	0.40	0.21
15	58431476	ALDH1A2, LIPC	rs1800588	high density lipoprotein cholesterol measurement	1	0.56	0.40	0.21
11	61802358	FADS2, FADS1	rs174546	high density lipoprotein cholesterol measurement, metabolic syndrome	1	0.02	0.57	0.35
20	35437976	GDF5	rs143384	hip bone size	1	.	.	.
20	35437976	GDF5	rs143384	hip circumference	1	.	.	.
20	35437976	GDF5	rs143384	hip circumference	1	.	.	.
14	22168978	TRAV30	rs11157436	HIV-1 infection, cognitive impairment measurement	1	0.06	0.61	0.19
2	169767711	KLHL23, PTCHD3P2	rs2114646	hormone measurement	1	0.02	0.43	0.21
16	89637957	DPEP1	rs1126464	hypertension	1	0.08	0.36	0.25
11	1884062	LSP1	rs661348	hypertension	1	0.14	0.59	0.43
19	10285007	ICAM1, AC011511.2	rs5498	ICAM-1 measurement	1	0.14	0.28	0.47
19	10285007	ICAM1, AC011511.2	rs5498	ICAM-1 measurement	1	0.14	0.28	0.47
19	10285007	ICAM1, AC011511.2	rs5498	ICAM-1 measurement	1	0.14	0.28	0.47
1	45331833	MUTYH, AL451136.1	rs3219489	immunoglobulin isotype switching measurement	1	0.26	0.39	0.24
20	35437976	GDF5	rs143384	infant body height	1	.	.	.
13	43883789	LACC1	rs3764147	inflammatory bowel disease	1	0.30	0.35	0.23
13	43883789	LACC1	rs3764147	inflammatory bowel disease	1	0.30	0.35	0.23
16	28609251	SULT1A1	rs750155	intelligence	1	0.34	0.45	0.44
16	28609251	SULT1A1	rs750155	intelligence	1	0.34	0.45	0.44
1	45331833	MUTYH, AL451136.1	rs3219489	intelligence	1	0.26	0.39	0.24
19	10285007	ICAM1, AC011511.2	rs5498	intercellular adhesion molecule 5 measurement	1	0.14	0.28	0.47

2	31388163	XDH	rs2073316	interleukin 18 measurement	1	0.34	0.28	0.46
3	169800667	LRRC34	rs6793295	interstitial lung disease	1	0.40	0.68	0.27
20	35437976	GDF5	rs143384	intertrochanteric region size	1	.	.	.
6	126643364	AL356534.1	rs4273712	intracranial volume measurement	1	0.05	0.44	0.28
2	26692756	KCNK3	rs12476527	Ischemic stroke	1	0.19	0.24	0.59
20	35437976	GDF5	rs143384	joint hypermobility measurement	1	.	.	.
20	35437976	GDF5	rs143384	Knee pain	1	.	.	.
20	35437976	GDF5	rs143384	lean body mass	1	.	.	.
20	35437976	GDF5	rs143384	lean body mass	1	.	.	.
20	35437976	GDF5	rs143384	lean body mass	1	.	.	.
13	43883789	LACC1	rs3764147	leprosy	1	0.30	0.35	0.23
8	141190368	DENND3	rs1045303	leukocyte count	1	0.20	0.62	0.26
12	6390367	LTBR	rs2286599	leukocyte count	1	0.07	0.03	0.16
19	51225221	CD33	rs12459419	leukocyte count	1	0.05	0.19	0.31
11	61803311	FADS1, FADS2	rs174547	linoleic acid measurement	1	0.02	0.57	0.35
11	61803311	FADS1, FADS2	rs174547	lipid measurement	1	0.02	0.57	0.35
11	61803311	FADS1, FADS2	rs174547	lipid measurement, blood metabolite measurement	1	0.02	0.57	0.35
6	46716485	PLA2G7	rs1805017	lipoprotein-associated phospholipase A(2) measurement	1	0.26	0.22	0.26
6	46716485	PLA2G7	rs1805017	lipoprotein-associated phospholipase A(2) measurement	1	0.26	0.22	0.26
3	111918969	PHLDB2	rs16858868	lobe attachment	1	0.05	0.67	0.35
11	61802358	FADS2, FADS1	rs174546	low density lipoprotein cholesterol measurement	1	0.02	0.57	0.35
11	61802358	FADS2, FADS1	rs174546	low density lipoprotein cholesterol measurement	1	0.02	0.57	0.35
11	61802358	FADS2, FADS1	rs174546	low density lipoprotein cholesterol measurement	1	0.02	0.57	0.35
1	62447248	USP1	rs10158897	low density lipoprotein cholesterol measurement	1	0.36	0.18	0.30
11	61781553	TMEM258, MYRF	rs174533	low density lipoprotein cholesterol measurement	1	0.03	0.57	0.35
11	61781553	TMEM258, MYRF	rs174533	low density lipoprotein cholesterol measurement	1	0.03	0.57	0.35
11	61781553	TMEM258, MYRF	rs174533	low density lipoprotein cholesterol measurement	1	0.03	0.57	0.35
11	61802358	FADS2, FADS1	rs174546	low density lipoprotein cholesterol measurement	1	0.02	0.57	0.35
19	11116926	LDLR	rs688	low density lipoprotein cholesterol measurement	1	0.04	0.18	0.44
19	11116926	LDLR	rs688	low density lipoprotein cholesterol measurement	1	0.04	0.18	0.44
20	35560231	FER1L4	rs224424	low density lipoprotein cholesterol measurement	1	.	.	.

1	62625187	DOCK7	rs6587980	low density lipoprotein cholesterol measurement	1	0.60	0.23	0.31
19	11116926	LDLR	rs688	low density lipoprotein cholesterol measurement	1	0.04	0.18	0.44
1	62584927	DOCK7	rs3850634	low density lipoprotein cholesterol measurement	1	0.39	0.18	0.30
11	61776489	TMEM258, MYRF	rs174529	low density lipoprotein cholesterol measurement	1	0.15	0.57	0.38
11	61802358	FADS2, FADS1	rs174546	low density lipoprotein cholesterol measurement	1	0.02	0.57	0.35
11	61803311	FADS1, FADS2	rs174547	low density lipoprotein cholesterol measurement	1	0.02	0.57	0.35
11	61803876	FADS1, FADS2	rs174548	low density lipoprotein cholesterol measurement	1	0.18	0.55	0.31
11	61811991	FADS1, FADS2	rs174554	low density lipoprotein cholesterol measurement	1	0.02	0.57	0.34
12	121012362	C12orf43	rs2264750	low density lipoprotein cholesterol measurement	1	0.11	0.47	0.32
11	61826344	FADS2, FADS1	rs174568	low density lipoprotein cholesterol measurement, physical activity	1	0.02	0.57	0.35
2	233416	SH3YL1	rs300756	low molecular weight phosphotyrosine protein phosphatase measurement	1	0.30	0.05	0.14
19	10285007	ICAM1, AC011511.2	rs5498	lymphocyte count	1	0.14	0.28	0.47
19	51225221	CD33	rs12459419	lymphocyte count	1	0.05	0.19	0.31
2	113196080	PSD4	rs4849169	lymphocyte count	1	0.18	0.65	0.47
19	51225221	CD33	rs12459419	lymphocyte count	1	0.05	0.19	0.31
22	41356743	ZC3H7B	rs12484074	lymphocyte count	1	0.05	0.08	0.24
19	10285007	ICAM1, AC011511.2	rs5498	lymphocyte count	1	0.14	0.28	0.47
2	113196080	PSD4	rs4849169	lymphocyte count	1	0.18	0.65	0.47
11	61803876	FADS1, FADS2	rs174548	lysophosphatidylcholine 20:5 measurement	1	0.18	0.55	0.31
11	61803876	FADS1, FADS2	rs174548	lysophosphatidylethanolamin e 20:4 measurement	1	0.18	0.55	0.31
4	174644094	GLRA3	rs4235212	Malignant epithelial tumor of ovary, response to paclitaxel	1	0.12	0.39	0.33
3	56737223	ARHGEF3	rs3772219	mean arterial pressure	1	0.57	0.42	0.29
11	65623846	PCNX3	rs12801636	mean arterial pressure	1	0.27	0.43	0.22
11	65617256	PCNX3	rs12790427	mean arterial pressure	1	0.26	0.42	0.20
11	1884062	LSP1	rs661348	mean arterial pressure	1	0.14	0.59	0.43
18	57571588	FECH	rs2272783	mean corpuscular hemoglobin	1	0.01	0.33	0.05
10	99529104	LINC01475	rs60386053	mean corpuscular hemoglobin	1	0.11	0.44	0.29
12	52077375	ATG101, AC025259.1	rs11521	mean corpuscular hemoglobin	1	0.07	0.44	0.22
18	57571588	FECH	rs2272783	mean corpuscular hemoglobin	1	0.01	0.33	0.05
18	57571588	FECH	rs2272783	mean corpuscular volume	1	0.01	0.33	0.05

17	76402433	UBE2O	rs11652985	mean corpuscular volume	1	0.03	0.20	0.45
18	57571588	FECH	rs2272783	mean corpuscular volume	1	0.01	0.33	0.05
7	151064793	SLC4A2	rs2303931	mean corpuscular volume	1	0.32	0.39	0.26
20	45796388	DNTTIP1	rs11477536	mean platelet volume	1	.	.	.
17	35557785	SLFN14	rs10512472	mean platelet volume	1	0.23	0.31	0.17
6	118894738	MCM9, ASF1A	rs14788658 8	mean platelet volume	1	0.06	0.38	0.20
6	118894738	MCM9, ASF1A	rs14788658 8	mean platelet volume	1	0.06	0.38	0.20
19	38359606	CATSPERG	rs60071299	mean platelet volume	1	0.21	0.60	0.30
17	35557785	SLFN14	rs10512472	mean platelet volume	1	0.23	0.31	0.17
11	61803311	FADS1, FADS2	rs174547	metabolite measurement	1	0.02	0.57	0.35
11	61803311	FADS1, FADS2	rs174547	metabolite measurement	1	0.02	0.57	0.35
5	35037010	AGXT2	rs37369	metabolite measurement	1	0.63	0.57	0.09
5	35037010	AGXT2	rs37369	metabolite measurement	1	0.63	0.57	0.09
19	44397392	ZNF285, AC245748.1, AC245748.2	rs2722651	methionine measurement	1	.	.	.
7	140024558	PARP12	rs2269996	monocyte count	1	0.33	0.53	0.20
6	160437156	SLC22A3	rs2292334	monocyte count	1	0.10	0.46	0.35
12	57449928	INHBC	rs2229357	monocyte count	1	0.08	0.07	0.19
9	136433917	INPP5E	rs10781543	monocyte percentage of leukocytes	1	0.41	0.34	0.40
3	36482913	STAC	rs2290530	mosquito bite reaction itch intensity measurement, mosquito bite reaction size measurement	1	0.09	0.31	0.11
3	169800667	LRRC34	rs6793295	multiple myeloma, monoclonal gammopathy	1	0.40	0.68	0.27
2	112013222	MERTK	rs57116599	multiple sclerosis	1	0.20	0.12	0.24
3	169800667	LRRC34	rs6793295	multiple sclerosis, chronic lymphocytic leukemia	1	0.40	0.68	0.27
19	51225221	CD33	rs12459419	myeloid cell surface antigen CD33 measurement	1	0.05	0.19	0.31
11	61812288	FADS1, FADS2	rs174555	myeloid white cell count	1	0.02	0.55	0.30
11	61803876	FADS1, FADS2	rs174548	myeloid white cell count	1	0.18	0.55	0.31
12	6390367	LTBR	rs2286599	myeloid white cell count	1	0.07	0.03	0.16
19	51225221	CD33	rs12459419	myeloid white cell count	1	0.05	0.19	0.31
7	140024558	PARP12	rs2269996	myeloid white cell count	1	0.33	0.53	0.20
11	61803876	FADS1, FADS2	rs174548	myeloid white cell count	1	0.18	0.55	0.31
12	6390367	LTBR	rs2286599	myeloid white cell count	1	0.07	0.03	0.16
4	71752606	GC	rs4588	myeloid white cell count	1	0.07	0.26	0.25
17	76402433	UBE2O	rs11652985	myeloid white cell count	1	0.03	0.20	0.45

19	51225221	CD33	rs12459419	myeloid white cell count	1	0.05	0.19	0.31
8	58411603	UBXN2B	rs2859998	narcolepsy with cataplexy	1	0.21	0.48	0.29
11	61783884	MYRF, TMEM258	rs174535	Nasal Cavity Polyp	1	0.15	0.57	0.35
19	36151982	COX7A1	rs17879437	neuritic plaque measurement	1	0.30	0.69	0.27
3	136435986	STAG1	rs66691851	neuroticism measurement	1	0.09	0.78	0.43
14	74911482	RPS6KL1	rs3213716	neuroticism measurement	1	0.02	0.58	0.41
22	40963782	RBX1	rs2413631	neuroticism measurement	1	0.02	0.03	0.25
14	41606749	AL121821.2 - LRFN5	rs1111179	neuroticism measurement	1	0.21	0.28	0.52
19	51225221	CD33	rs12459419	neutrophil count	1	0.05	0.19	0.31
19	51225221	CD33	rs12459419	neutrophil count	1	0.05	0.19	0.31
1	36461929	MRPS15	rs78245381	neutrophil count	1	0.16	0.32	0.33
11	61812288	FADS1, FADS2	rs174555	neutrophil count, eosinophil count	1	0.02	0.55	0.30
3	169769713	AC078802.1	rs9811216	neutrophil percentage of granulocytes	1	0.30	0.68	0.26
11	61803311	FADS1, FADS2	rs174547	omega-3 polyunsaturated fatty acid measurement, metabolic syndrome	1	0.02	0.57	0.35
11	61803311	FADS1, FADS2	rs174547	omega-6 polyunsaturated fatty acid measurement	1	0.02	0.57	0.35
11	61803311	FADS1, FADS2	rs174547	omega-6 polyunsaturated fatty acid measurement	1	0.02	0.57	0.35
11	61812288	FADS1, FADS2	rs174555	omega-6 polyunsaturated fatty acid measurement	1	0.02	0.55	0.30
11	61803311	FADS1, FADS2	rs174547	omega-6:omega-3 polyunsaturated fatty acid ratio, metabolic syndrome	1	0.02	0.57	0.35
8	127452704	CASC8	rs58376190	opioid dependence, methadone dose measurement	1	0.57	0.51	0.21
16	89637957	DPEP1	rs1126464	osteoarthritis	1	0.08	0.36	0.25
20	35437976	GDF5	rs143384	osteoarthritis, knee	1	.	.	.
20	35437976	GDF5	rs143384	osteoarthritis, knee	1	.	.	.
1	111649500	KRT18P57, RAP1A	rs494453	osteoporosis	1	0.47	0.47	0.38
1	113905767	DCLRE1B	rs11552449	parental longevity	1	0.02	0.59	0.19
5	134863415	AC006077.1	rs11950533	Parkinson's disease	1	0.04	0.31	0.14
4	61587491	ADGRL3	rs2172802	partial epilepsy	1	0.44	0.62	0.28
20	35437976	GDF5	rs143384	peak expiratory flow	1	.	.	.
9	136362314	CARD9, DNLZ	rs3829109	peak insulin response measurement	1	0.15	0.05	0.28
11	61803876	FADS1, FADS2	rs174548	phosphatidylcholine 32:0 measurement	1	0.18	0.55	0.31
11	61783884	MYRF, TMEM258	rs174535	phosphatidylcholine 38:5 measurement	1	0.15	0.57	0.35
11	61783884	MYRF, TMEM258	rs174535	phosphatidylcholine 38:6 measurement	1	0.15	0.57	0.35

11	61783884	MYRF, TMEM258	rs174535	phosphatidylcholine 40:6 measurement	1	0.15	0.57	0.35
11	61776027	MYRF, TMEM258	rs174528	phosphatidylcholine ether measurement	1	0.45	0.57	0.39
11	61803876	FADS1, FADS2	rs174548	phospholipid measurement	1	0.18	0.55	0.31
1	21617327	RAP1GAP	rs2275360	phosphorus measurement	1	0.19	0.53	0.25
3	141576936	RASA2	rs3732869	physical activity measurement, body mass index	1	0.25	0.21	0.06
3	141576936	RASA2	rs3732869	physical activity measurement, body mass index	1	0.25	0.21	0.06
19	47093845	ZC3H4	rs10408163	physical activity measurement, body mass index	1	0.10	0.27	0.69
19	47093845	ZC3H4	rs10408163	physical activity measurement, body mass index	1	0.10	0.27	0.69
19	47093845	ZC3H4	rs10408163	physical activity measurement, body mass index	1	0.10	0.27	0.69
17	35596588	AP2B1	rs11653357	platelet component distribution width	1	0.30	0.31	0.17
17	35557785	SLFN14	rs10512472	platelet count	1	0.23	0.31	0.17
6	33574746	BAK1, GGNBP1	rs9296095	platelet count	1	0.33	0.24	0.22
17	35596588	AP2B1	rs11653357	platelet count	1	0.30	0.31	0.17
11	61803876	FADS1, FADS2	rs174548	platelet count	1	0.18	0.55	0.31
1	36307804	SH3D21	rs35343437	platelet count	1	0.12	0.72	0.44
1	36307804	SH3D21	rs35343437	platelet count	1	0.12	0.72	0.44
6	33574746	BAK1, GGNBP1	rs9296095	platelet count	1	0.33	0.24	0.22
19	55181876	PTPRH	rs2288419	platelet count	1	0.47	0.44	0.19
19	51225221	CD33	rs12459419	platelet crit	1	0.05	0.19	0.31
19	18542034	FKBP8	rs10854166	potassium measurement	1	0.03	0.41	0.49
17	39863888	IKZF3	rs9635726	primary biliary cirrhosis	1	0.02	0.64	0.19
20	49905793	SPATA2	rs495337	psoriasis	1	0.10	0.34	0.40
20	49905793	SPATA2	rs495337	psoriasis	1	0.10	0.34	0.40
20	49905793	SPATA2	rs495337	psoriasis	1	0.10	0.34	0.40
1	27382530	CD164L2	rs2504779	psoriasis	1	.	.	.
5	139481493	STING1	rs11554776	psoriasis	1	0.01	0.40	0.15
5	148476770	HTR4	rs7733088	pulmonary function measurement	1	0.29	0.64	0.37
11	1884062	LSP1	rs661348	pulse pressure measurement	1	0.14	0.59	0.43
2	26692756	KCNK3	rs12476527	pulse pressure measurement	1	0.19	0.24	0.59
4	15963240	FGFBP2, PROM1	rs4698433	QT interval	1	0.13	0.28	0.36

11	61802358	FADS2, FADS1	rs174546	QT interval	1	0.02	0.57	0.35
2	200306468	SPATS2L	rs10931898	reaction time measurement	1	0.15	0.73	0.38
17	39972512	GSDMA	rs8077456	reaction time measurement	1	0.24	0.40	0.34
14	32824325	AKAP6	rs4647899	reaction time measurement	1	0.20	0.34	0.28
11	61781087	MYRF, TMEM258	rs509360	red blood cell density measurement	1	0.08	0.57	0.71
16	10532030	AC027277.1, EMP2	rs7195563	red blood cell density measurement	1	0.22	0.60	0.52
18	57571588	FECH	rs2272783	red blood cell distribution width	1	0.01	0.33	0.05
1	161233437	NR1I3	rs2502815	red blood cell distribution width	1	0.36	0.44	0.25
18	57571588	FECH	rs2272783	red blood cell distribution width	1	0.01	0.33	0.05
19	51225221	CD33	rs12459419	red blood cell distribution width	1	0.05	0.19	0.31
18	57571588	FECH	rs2272783	red blood cell distribution width	1	0.01	0.33	0.05
11	61783884	MYRF, TMEM258	rs174535	refractive error measurement	1	0.15	0.57	0.35
15	53615751	WDR72	rs17730281	renal system measurement, blood urea nitrogen measurement	1	0.12	0.49	0.20
11	61783884	MYRF, TMEM258	rs174535	respiratory system disease	1	0.15	0.57	0.35
12	62114243	TAFA2	rs1985875	response to bronchodilator, chronic obstructive pulmonary disease, FEV/FEC ratio	1	0.11	0.36	0.26
15	78580777	CHRNA5	rs569207	response to bronchodilator, FEV/FEC ratio	1	0.32	0.48	0.24
15	78620601	CHRNA3	rs7170068	response to bronchodilator, FEV/FEC ratio	1	0.23	0.47	0.23
16	84762997	USP10	rs12932018	response to mTOR inhibitor	1	0.05	0.41	0.25
11	123058167	HSPA8	rs4936770	response to tenofovir, HIV infection, creatinine clearance measurement	1	0.57	0.55	0.22
11	123057914	HSPA8	rs4802	response to tenofovir, HIV infection, creatinine clearance measurement	1	0.57	0.55	0.22
11	123058167	HSPA8	rs4936770	response to tenofovir, HIV infection, creatinine clearance measurement	1	0.57	0.55	0.22
11	61803311	FADS1, FADS2	rs174547	resting heart rate	1	0.02	0.57	0.35
1	39525916	BMP8A, PPIEL	rs3738676	resting heart rate, chronic obstructive pulmonary disease	1	0.36	0.44	0.37
8	141218810	SLC45A4	rs753778	reticulocyte count	1	0.37	0.40	0.29
17	76385474	SPHK1	rs2247856	reticulocyte count	1	0.04	0.19	0.45
8	141218810	SLC45A4	rs753778	reticulocyte count	1	0.37	0.40	0.29
8	141218810	SLC45A4	rs753778	reticulocyte count	1	0.37	0.40	0.29

17	76385474	SPHK1	rs2247856	reticulocyte count	1	0.04	0.19	0.45
1	61277488	NFIA	rs332827	risk-taking behaviour	1	0.03	0.47	0.45
7	1874841	AC069288.1, MAD1L1	rs10950415	risky sexual behaviour measurement	1	0.28	0.44	0.42
3	136435986	STAG1	rs66691851	schizophrenia	1	0.09	0.78	0.43
3	136435986	STAG1	rs66691851	schizophrenia	1	0.09	0.78	0.43
3	136435986	STAG1	rs66691851	schizophrenia	1	0.09	0.78	0.43
3	136435986	STAG1	rs66691851	schizophrenia	1	0.09	0.78	0.43
3	136435986	STAG1	rs66691851	schizophrenia	1	0.09	0.78	0.43
3	53225980	TKT	rs3816767	self reported educational attainment	1	0.21	0.60	0.46
7	24716758	GSDME	rs2299098	self reported educational attainment	1	0.46	0.37	0.18
16	690404	WDR24, Z92544.1	rs4984682	self reported educational attainment	1	0.43	0.72	0.26
6	118894238	ASF1A, MCM9	rs11542663	self reported educational attainment	1	0.15	0.65	0.37
1	163172765	AL499616.1, RGS5	rs12035879	serum alanine aminotransferase measurement	1	0.11	0.40	0.37
5	35037010	AGXT2	rs37369	serum dimethylarginine measurement	1	0.63	0.57	0.09
5	35037010	AGXT2	rs37369	serum dimethylarginine measurement	1	0.63	0.57	0.09
20	38349567	LBP	rs1739654	serum IgG glycosylation measurement	1	.	.	.
11	61803311	FADS1, FADS2	rs174547	serum metabolite measurement	1	0.02	0.57	0.35
11	61803876	FADS1, FADS2	rs174548	serum metabolite measurement	1	0.18	0.55	0.31
11	61803311	FADS1, FADS2	rs174547	serum metabolite measurement	1	0.02	0.57	0.35
11	61803311	FADS1, FADS2	rs174547	serum metabolite measurement	1	0.02	0.57	0.35
11	61783884	MYRF, TMEM258	rs174535	serum metabolite measurement	1	0.15	0.57	0.35
11	61802358	FADS2, FADS1	rs174546	serum metabolite measurement	1	0.02	0.57	0.35
11	61803311	FADS1, FADS2	rs174547	serum metabolite measurement	1	0.02	0.57	0.35
11	61802358	FADS2, FADS1	rs174546	serum metabolite measurement	1	0.02	0.57	0.35
15	58431476	ALDH1A2, LIPC	rs1800588	serum metabolite measurement	1	0.56	0.40	0.21
11	61802358	FADS2, FADS1	rs174546	serum metabolite measurement	1	0.02	0.57	0.35
11	61803311	FADS1, FADS2	rs174547	serum metabolite measurement	1	0.02	0.57	0.35
15	58431476	ALDH1A2, LIPC	rs1800588	serum metabolite measurement	1	0.56	0.40	0.21
15	58431476	ALDH1A2, LIPC	rs1800588	serum metabolite measurement	1	0.56	0.40	0.21
11	61802358	FADS2, FADS1	rs174546	serum metabolite measurement	1	0.02	0.57	0.35

11	61802358	FADS2, FADS1	rs174546	serum metabolite measurement	1	0.02	0.57	0.35
15	58431476	ALDH1A2, LIPC	rs1800588	serum metabolite measurement	1	0.56	0.40	0.21
15	58431476	ALDH1A2, LIPC	rs1800588	serum metabolite measurement	1	0.56	0.40	0.21
15	58431476	ALDH1A2, LIPC	rs1800588	serum metabolite measurement	1	0.56	0.40	0.21
11	61803876	FADS1, FADS2	rs174548	serum metabolite measurement	1	0.18	0.55	0.31
11	61803311	FADS1, FADS2	rs174547	serum metabolite measurement	1	0.02	0.57	0.35
11	61792609	TMEM258	rs174538	serum metabolite measurement	1	0.02	0.56	0.31
11	61803311	FADS1, FADS2	rs174547	serum metabolite measurement	1	0.02	0.57	0.35
11	61802358	FADS2, FADS1	rs174546	serum metabolite measurement	1	0.02	0.57	0.35
15	58431476	ALDH1A2, LIPC	rs1800588	serum metabolite measurement	1	0.56	0.40	0.21
11	61779765	MYRF, TMEM258	rs108499	serum metabolite measurement	1	0.02	0.56	0.33
11	61783884	MYRF, TMEM258	rs174535	serum metabolite measurement	1	0.15	0.57	0.35
15	58431476	ALDH1A2, LIPC	rs1800588	serum metabolite measurement	1	0.56	0.40	0.21
11	61802358	FADS2, FADS1	rs174546	serum metabolite measurement	1	0.02	0.57	0.35
15	58431476	ALDH1A2, LIPC	rs1800588	serum metabolite measurement	1	0.56	0.40	0.21
11	61802358	FADS2, FADS1	rs174546	serum metabolite measurement	1	0.02	0.57	0.35
11	61802358	FADS2, FADS1	rs174546	serum metabolite measurement	1	0.02	0.57	0.35
11	61802358	FADS2, FADS1	rs174546	serum metabolite measurement	1	0.02	0.57	0.35
11	61783884	MYRF, TMEM258	rs174535	serum metabolite measurement	1	0.15	0.57	0.35
16	89919746	AC092143.1, MC1R	rs885479	skin pigmentation measurement	1	0.01	0.62	0.07
15	58431476	ALDH1A2, LIPC	rs1800588	sleep duration, high density lipoprotein cholesterol measurement	1	0.56	0.40	0.21
15	58431476	ALDH1A2, LIPC	rs1800588	sleep duration, high density lipoprotein cholesterol measurement	1	0.56	0.40	0.21
11	61790354	TMEM258	rs102274	sleep duration, low density lipoprotein cholesterol measurement	1	0.03	0.57	0.35
11	116830406	APOC3	rs2070669	sleep duration, triglyceride measurement	1	0.12	0.53	0.68
11	116830406	APOC3	rs2070669	sleep duration, triglyceride measurement	1	0.12	0.53	0.68
5	36694954	AC008957.1	rs13171357	smoking behavior, unipolar depression	1	0.11	0.31	0.28
16	696611	FBXL16	rs11861214	smoking behaviour measurement	1	0.21	0.71	0.26

16	670986	RHOT2	rs1139897	smoking initiation	1	0.04	0.68	0.26
11	113789854	ATF4P4, AP003170.2	rs1713676	smoking status measurement	1	0.16	0.42	0.56
16	670986	RHOT2	rs1139897	smoking status measurement	1	0.04	0.68	0.26
11	1884062	LSP1	rs661348	smoking status measurement, diastolic blood pressure	1	0.14	0.59	0.43
11	1884062	LSP1	rs661348	smoking status measurement, systolic blood pressure	1	0.14	0.59	0.43
11	61803311	FADS1, FADS2	rs174547	sphingolipid measurement, blood metabolite measurement	1	0.02	0.57	0.35
11	61803311	FADS1, FADS2	rs174547	sphingolipid measurement, blood metabolite measurement	1	0.02	0.57	0.35
20	35437976	GDF5	rs143384	spine bone size	1	.	.	.
11	61803311	FADS1, FADS2	rs174547	spine bone size	1	0.02	0.57	0.35
2	26692756	KCNK3	rs12476527	stroke	1	0.19	0.24	0.59
10	109875454	XPNPEP1	rs3818285	superior crus of antihelix expression	1	0.16	0.39	0.24
12	128809788	SLC15A4	rs11059927	systemic lupus erythematosus	1	0.00	0.19	0.11
8	8240557	FAM86B3P, FAM86B3P, ALG1L13P, ALG1L13P	rs2955587	systemic lupus erythematosus	1	0.05	0.81	0.43
17	39965740	GSDMA	rs3894194	systemic scleroderma	1	0.26	0.54	0.45
16	89637957	DPEP1	rs1126464	systolic blood pressure	1	0.08	0.36	0.25
11	1884062	LSP1	rs661348	systolic blood pressure	1	0.14	0.59	0.43
11	65617256	PCNX3	rs12790427	systolic blood pressure	1	0.26	0.42	0.20
4	2696637	FAM193A	rs3733215	systolic blood pressure	1	0.23	0.40	0.40
11	1884062	LSP1	rs661348	systolic blood pressure	1	0.14	0.59	0.43
2	26692756	KCNK3	rs12476527	systolic blood pressure	1	0.19	0.24	0.59
2	26709928	KCNK3	rs1275923	systolic blood pressure, alcohol drinking	1	0.14	0.24	0.59
3	169800667	LRRC34	rs6793295	thyroid carcinoma	1	0.40	0.68	0.27
11	61802358	FADS2, FADS1	rs174546	total cholesterol measurement	1	0.02	0.57	0.35
11	61802358	FADS2, FADS1	rs174546	total cholesterol measurement	1	0.02	0.57	0.35
11	61811991	FADS1, FADS2	rs174554	total cholesterol measurement	1	0.02	0.57	0.34
12	121012362	C12orf43	rs2264750	total cholesterol measurement	1	0.11	0.47	0.32
15	58431476	ALDH1A2, LIPC	rs1800588	total cholesterol measurement	1	0.56	0.40	0.21
15	58431280	LIPC, ALDH1A2	rs1077834	total cholesterol measurement	1	0.57	0.42	0.21
11	61781553	TMEM258, MYRF	rs174533	total cholesterol measurement	1	0.03	0.57	0.35
15	58431476	ALDH1A2, LIPC	rs1800588	total cholesterol measurement	1	0.56	0.40	0.21

11	61803876	FADS1, FADS2	rs174548	total cholesterol measurement	1	0.18	0.55	0.31
11	61811991	FADS1, FADS2	rs174554	total cholesterol measurement	1	0.02	0.57	0.34
11	61776489	TMEM258, MYRF	rs174529	total cholesterol measurement	1	0.15	0.57	0.38
11	61802358	FADS2, FADS1	rs174546	total cholesterol measurement	1	0.02	0.57	0.35
11	61803311	FADS1, FADS2	rs174547	total cholesterol measurement	1	0.02	0.57	0.35
1	62584927	DOCK7	rs3850634	total cholesterol measurement	1	0.39	0.18	0.30
1	62625187	DOCK7	rs6587980	total cholesterol measurement	1	0.60	0.23	0.31
19	11116926	LDLR	rs688	total cholesterol measurement	1	0.04	0.18	0.44
11	61803311	FADS1, FADS2	rs174547	total cholesterol measurement, diastolic blood pressure, triglyceride measurement, systolic blood pressure, hematocrit, ventricular rate measurement, glucose measurement, body mass index, high density lipoprotein cholesterol measurement	1	0.02	0.57	0.35
11	61803311	FADS1, FADS2	rs174547	total cholesterol measurement, hematocrit, stroke, ventricular rate measurement, body mass index, atrial fibrillation, high density lipoprotein cholesterol measurement, coronary artery disease, cancer, diastolic blood pressure, triglyceride measurement, systolic blood pressure, heart failure, diabetes mellitus, glucose measurement, mortality	1	0.02	0.57	0.35
11	61776489	TMEM258, MYRF	rs174529	total iron binding capacity	1	0.15	0.57	0.38
11	61776489	TMEM258, MYRF	rs174529	total iron binding capacity	1	0.15	0.57	0.38
11	61803311	FADS1, FADS2	rs174547	triacylglycerol 52:2 measurement	1	0.02	0.57	0.35
11	61783884	MYRF, TMEM258	rs174535	triacylglycerol 56:7 measurement	1	0.15	0.57	0.35
11	61803876	FADS1, FADS2	rs174548	triacylglycerol 58:10 measurement	1	0.18	0.55	0.31
11	61813163	FADS1, FADS2	rs174556	triacylglycerol 58:10 measurement	1	0.02	0.55	0.30
11	61783884	MYRF, TMEM258	rs174535	triacylglycerol 58:9 measurement	1	0.15	0.57	0.35
11	61803311	FADS1, FADS2	rs174547	triglyceride measurement	1	0.02	0.57	0.35

11	61803876	FADS1, FADS2	rs174548	triglyceride measurement	1	0.18	0.55	0.31
11	61802358	FADS2, FADS1	rs174546	triglyceride measurement	1	0.02	0.57	0.35
5	157037231	HAVCR1	rs2036402	triglyceride measurement	1	0.04	0.12	0.29
11	61802358	FADS2, FADS1	rs174546	triglyceride measurement	1	0.02	0.57	0.35
15	58431227	ALDH1A2, LIPC	rs1077835	triglyceride measurement	1	0.57	0.42	0.21
11	61776489	TMEM258, MYRF	rs174529	triglyceride measurement	1	0.15	0.57	0.38
15	58431476	ALDH1A2, LIPC	rs1800588	triglyceride measurement	1	0.56	0.40	0.21
15	58431476	ALDH1A2, LIPC	rs1800588	triglyceride measurement	1	0.56	0.40	0.21
15	58431476	ALDH1A2, LIPC	rs1800588	triglyceride measurement	1	0.56	0.40	0.21
15	58431476	ALDH1A2, LIPC	rs1800588	triglyceride measurement	1	0.56	0.40	0.21
11	61802358	FADS2, FADS1	rs174546	triglyceride measurement	1	0.02	0.57	0.35
1	62584927	DOCK7	rs3850634	triglyceride measurement	1	0.39	0.18	0.30
1	62625187	DOCK7	rs6587980	triglyceride measurement	1	0.60	0.23	0.31
11	61776489	TMEM258, MYRF	rs174529	triglyceride measurement	1	0.15	0.57	0.38
11	61802358	FADS2, FADS1	rs174546	triglyceride measurement	1	0.02	0.57	0.35
11	61803311	FADS1, FADS2	rs174547	triglyceride measurement	1	0.02	0.57	0.35
11	61803876	FADS1, FADS2	rs174548	triglyceride measurement	1	0.18	0.55	0.31
11	61811991	FADS1, FADS2	rs174554	triglyceride measurement	1	0.02	0.57	0.34
15	58431476	ALDH1A2, LIPC	rs1800588	triglyceride measurement	1	0.56	0.40	0.21
11	61802358	FADS2, FADS1	rs174546	triglyceride measurement	1	0.02	0.57	0.35
13	113821971	GAS6, GAS6-AS1	rs7400002	triglyceride measurement	1	0.52	0.40	0.24
15	58431227	ALDH1A2, LIPC	rs1077835	triglyceride measurement	1	0.57	0.42	0.21
15	58431280	LIPC, ALDH1A2	rs1077834	triglyceride measurement	1	0.57	0.42	0.21
15	58431227	ALDH1A2, LIPC	rs1077835	triglyceride measurement	1	0.57	0.42	0.21
15	58431227	ALDH1A2, LIPC	rs1077835	triglyceride measurement	1	0.57	0.42	0.21
15	58431227	ALDH1A2, LIPC	rs1077835	triglyceride measurement	1	0.57	0.42	0.21
13	94596312	TGDS - GPR180	rs2298058	triglyceride measurement	1	0.02	0.59	0.32
11	61802358	FADS2, FADS1	rs174546	triglyceride measurement, C-reactive protein measurement	1	0.02	0.57	0.35
20	35437976	GDF5	rs143384	trochanter size	1	.	.	.

6	126643364	AL356534.1	rs4273712	type II diabetes mellitus	1	0.05	0.44	0.28
17	3924792	ATP2A3	rs1043246	type II diabetes mellitus	1	0.01	0.47	0.15
12	71129173	TSPAN8	rs1796330	type II diabetes mellitus	1	0.42	0.30	0.42
8	144778182	ZNF34	rs2294120	type II diabetes mellitus	1	0.05	0.74	0.44
6	126643364	AL356534.1	rs4273712	type II diabetes mellitus	1	0.05	0.44	0.28
5	35037010	AGXT2	rs37369	urinary metabolite measurement	1	0.63	0.57	0.09
5	35037010	AGXT2	rs37369	urinary metabolite measurement	1	0.63	0.57	0.09
5	35037010	AGXT2	rs37369	urinary metabolite measurement	1	0.63	0.57	0.09
5	35037010	AGXT2	rs37369	urinary metabolite measurement	1	0.63	0.57	0.09
5	35037010	AGXT2	rs37369	urinary metabolite measurement	1	0.63	0.57	0.09
5	35037010	AGXT2	rs37369	urinary metabolite measurement	1	0.63	0.57	0.09
5	35037010	AGXT2	rs37369	urinary metabolite measurement	1	0.63	0.57	0.09
5	35037010	AGXT2	rs37369	urinary metabolite measurement	1	0.63	0.57	0.09
5	35037010	AGXT2	rs37369	urinary metabolite measurement	1	0.63	0.57	0.09
11	61776027	MYRF, TMEM258	rs174528	vaccenic acid measurement	1	0.45	0.57	0.39
11	61776027	MYRF, TMEM258	rs174528	vaccenic acid measurement	1	0.45	0.57	0.39
4	71752606	GC	rs4588	vitamin D measurement	1	0.07	0.26	0.25
4	71752606	GC	rs4588	vitamin D measurement	1	0.07	0.26	0.25
4	71752606	GC	rs4588	vitamin D measurement	1	0.07	0.26	0.25
15	58431476	ALDH1A2, LIPC	rs1800588	vitamin D measurement	1	0.56	0.40	0.21
22	40273644	TNRC6B	rs733381	waist-hip ratio	1	0.06	0.29	0.20
20	35437976	GDF5	rs143384	waist-hip ratio	1	.	.	.
20	35437976	GDF5	rs143384	waist-hip ratio	1	.	.	.
21	46122306	COL6A2	rs2070578	waist-hip ratio	1	0.16	0.43	0.51
9	128722247	ZDHHC12	rs10988105	waist-hip ratio	1	0.05	0.11	0.50
14	74670961	AREL1	rs2302832	wellbeing measurement	1	0.03	0.54	0.53
19	35738053	KMT2B	rs2242522	white matter microstructure measurement	1	0.15	0.50	0.51
22	40963782	RBX1	rs2413631	worry measurement	1	0.02	0.03	0.25
3	136435986	STAG1	rs66691851	worry measurement	1	0.09	0.78	0.43
